# Zika virus infection at mid-gestation results in fetal cerebral cortical injury and fetal death in the olive baboon

**DOI:** 10.1371/journal.ppat.1007507

**Published:** 2019-01-18

**Authors:** Sunam Gurung, Nicole Reuter, Alisha Preno, Jamie Dubaut, Hugh Nadeau, Kimberly Hyatt, Krista Singleton, Ashley Martin, W. Tony Parks, James F. Papin, Dean A. Myers

**Affiliations:** 1 Department of Obstetrics and Gynecology, University of Oklahoma Health Sciences Center, Oklahoma City, OK, United States of America; 2 Division of Comparative Medicine, Department of Pathology, University of Oklahoma Health Sciences Center, Oklahoma City, Oklahoma, United States of America; 3 Department of Physiology, University of Oklahoma Health Sciences Center, Oklahoma City, Oklahoma, United States of America; 4 Department of Pathology, University of Toronto, Toronto, Ontario, Canada; University of Pittsburgh, UNITED STATES

## Abstract

Zika virus (ZIKV) infection during pregnancy in humans is associated with an increased incidence of congenital anomalies including microcephaly as well as fetal death and miscarriage and collectively has been referred to as Congenital Zika Syndrome (CZS). Animal models for ZIKV infection in pregnancy have been developed including mice and non-human primates (NHPs). In macaques, fetal CZS outcomes from maternal ZIKV infection range from none to significant. In the present study we develop the olive baboon (*Papio anubis*), as a model for vertical transfer of ZIKV during pregnancy. Four mid-gestation, timed-pregnant baboons were inoculated with the French Polynesian ZIKV isolate (10^4^ ffu). This study specifically focused on the acute phase of vertical transfer. Dams were terminated at 7 days post infection (dpi; n = 1), 14 dpi (n = 2) and 21 dpi (n = 1). All dams exhibited mild to moderate rash and conjunctivitis. Viremia peaked at 5–7 dpi with only one of three dams remaining mildly viremic at 14 dpi. An anti-ZIKV IgM response was observed by 14 dpi in all three dams studied to this stage, and two dams developed a neutralizing IgG response by either 14 dpi or 21 dpi, the latter included transfer of the IgG to the fetus (cord blood). A systemic inflammatory response (increased IL2, IL6, IL7, IL15, IL16) was observed in three of four dams. Vertical transfer of ZIKV to the placenta was observed in three pregnancies (n = 2 at 14 dpi and n = 1 at 21 dpi) and ZIKV was detected in fetal tissues in two pregnancies: one associated with fetal death at ~14 dpi, and the other in a viable fetus at 21 dpi. ZIKV RNA was detected in the fetal cerebral cortex and other tissues of both of these fetuses. In the fetus studied at 21 dpi with vertical transfer of virus to the CNS, the frontal cerebral cortex exhibited notable defects in radial glia, radial glial fibers, disorganized migration of immature neurons to the cortical layers, and signs of pathology in immature oligodendrocytes. In addition, indices of pronounced neuroinflammation were observed including astrogliosis, increased microglia and IL6 expression. Of interest, in one fetus examined at 14 dpi without detection of ZIKV RNA in brain and other fetal tissues, increased neuroinflammation (IL6 and microglia) was observed in the cortex. Although the placenta of the 14 dpi dam with fetal death showed considerable pathology, only minor pathology was noted in the other three placentas. ZIKV was detected immunohistochemically in two placentas (14 dpi) and one placenta at 21 dpi but not at 7 dpi. This is the first study to examine the early events of vertical transfer of ZIKV in a NHP infected at mid-gestation. The baboon thus represents an additional NHP as a model for ZIKV induced brain pathologies to contrast and compare to humans as well as other NHPs.

## Author summary

Zika virus is endemic in the Americas, primarily spread through mosquitos and sexual contact. Zika virus infection during pregnancy in women is associated with a variety of fetal pathologies now referred to as Congenital Zika Syndrome (CZS), with the most severe pathology being fetal microcephaly. Developing model organisms that faithfully recreate Zika infection in humans is critical for future development of treatments and preventions. In our present study, we infected Olive baboons at mid-gestation with Zika virus and studied the acute period of viremia and transfer of Zika virus to the fetus during the first three weeks after infection to better understand the timing and mechanisms of transfer of ZIKV across the placenta, leading to CZS. We observed Zika virus transfer to fetuses resulting in fetal death in one pregnancy and in a second pregnancy, significant damage to the frontal cortex of the fetal brain at a critical period of neurodevelopment in primates. Thus, the baboon provides a promising new non-human primate model to further compare and contrast the consequences of Zika virus infection in pregnancy to humans and other non-human primates.

## Introduction

Originally isolated from a febrile sentinel rhesus monkey in the Zika forest in Uganda in 1947, Zika virus (ZIKV) belongs to the *Flaviviridae* family, genus Flavivirus, which includes dengue (DENV), West Nile (WNV), yellow fever (YFV), and Japanese encephalitis virus (JEV) [1, 2]. Zika virus infection during pregnancy has now been firmly linked to an increased incidence of newborns with microcephaly and a variety of other congenital anomalies, collectively referred to as Congenital Zika Syndrome (CZS) [3–6]. It was recently shown that one in seven children born from women with confirmed or possible ZIKV infection during gestation in Puerto Rico had a birth defect or neurodevelopmental abnormality. [7] In addition to the spectrum of congenital malformations, ZIKV infection in pregnancy is associated with intrauterine fetal demise and increased incidence of miscarriage [3, 4].

The development of animal models that faithfully recapitulate the complex pathogenesis of ZIKV infection including trans-placental passage of the virus resulting in CZS anomalies is essential for developing and testing vaccines and anti-viral strategies. Although mice have been widely used to study ZIKV infection and fetal outcome, in order for pregnant mice to be infected with ZIKV, interferon (IFN) signaling must be blocked raising questions regarding the translational application of findings to humans [8–10]. Alternatively, fetal ZIKV infection in mice has been achieved via direct viral inoculation of the fetus, neonate or uterus/placenta [11–15]. These studies verified that ZIKV infection results in a range of fetal pathologies including fetal demise, intrauterine growth restriction and fetal CNS pathologies. While mouse models have provided insight into ZIKV pathogenesis, non-human primates (NHPs) are the best-documented animal reservoirs for Zika and related flaviviruses. ZIKV infection has been characterized in male and non-pregnant female rhesus macaques (*Macacca mulatta; [16–22])* cynomolgus macaques (*Macacca fascicularis; [23, 24])* and baboons (*Papio anubis*; [25]) following the standard subcutaneous (sc) route of inoculation. Successful infection of rhesus macaques has also been described following intra-vaginal/intra-rectal [26, 27], oropharangeal mucosal [28] or mosquito bite [29] routes of inoculation, and seroprevalence of ZIKV has been reported in wild African Green Monkeys (*Chlorocebus aethiops*) and baboons [30].

Zika virus infection of pregnant rhesus macaques [16, 31–36], pigtail macaques (*Macacca nemestrina; [37, 38]*), and marmosets (*Callithrix jacchus; [39]),* has been achieved to model pregnancy outcomes and feto-placental pathologies in NHPs. Similar to humans, intrauterine fetal death and/or miscarriage has been reported as a common (26%) outcome following ZIKV infection in macaques [40]. While microcephaly has not been reported in macaques infected with ZIKV during gestation, a range of fetal or infant neuropathologies has been documented. An initial study of four rhesus macaques, sc inoculation (mid-1^st^ or early 3^rd^ trimesters) with the French Polynesian strain of ZIKV [31], resulted in no overt fetal brain pathology by late gestation, although ocular and lung pathology was observed. Subsequently [32], only subtle effects on fetal brain structure were noted in 2/5 fetuses from pregnant rhesus macaques following sc inoculation (1^st^ or 2^nd^ trimesters) with the Puerto Rican strain of ZIKV. However, significant fetal neuropathology was reported in a single pigtail macaque following multi-site sc infection [37] with the Cambodian strain of ZIKV, albeit the dose of ZIKV was artificially high (5x10^7^ pfu). In this study, the most consistent CNS pathology was loss of fetal non-cortical brain volume with white matter and ependymal epithelium injury with gliosis. However, the authors noted normal cortical folding and found no evidence of cortical malformations. A subsequent study from this group using the same inoculating dose of either the Cambodian (2 macaques) or Brazilian ZIKV strain (three macaques) confirmed the initial findings and additionally reported a decrease in late neuroprogenitor cells (NPCs) in the subgranular zone (SGZ) of the hippocampal dentate gyrus and subventricular zone (SVZ) of the temporal cortex [38]. Interestingly, the Cambodian ZIKV strain is not associated with adverse pregnancy outcome or CZS in humans. The most severe fetal neuropathology in NHPs was recently reported in a study of six rhesus macaques following sc inoculation with the Brazilian strain of ZIKV (1x10^3^ pfu) early in pregnancy which resulted in one *in utero* fetal death/abortion while the remaining five infants (at birth) exhibited smaller brain size and CNS lesions including calcifications, hemorrhage, necrosis, vasculitis, gliosis and apoptosis of NPCs [34]. In addition, these authors found significant placental pathology, potentially contributing to the more extensive infant CNS pathology observed in this study. Another study of four pregnant rhesus macaques (late 1^st^ to late 2^nd^ trimester) circumvented the need for vertical transfer by simultaneous ZIKV (Brazilian strain) inoculation via both intra-amniotic and maternal intravenous routes [33]. These authors noted reduced NPCs in the SGZ of the hippocampal dentate gyrus but not in the cortical SVZ coupled with areas of calcification and gliosis. Subcutaneous inoculation of two marmosets with ZIKV resulted *in utero* fetal death and miscarriage at 16–18 dpi. Although fetal CNS pathology was observed in one fetal marmoset, it is not clear if this was in response to ZIKV or as an outcome of *in utero* fetal death [39]. Cumulatively, these studies confirm that macaques have a high (100%) rate of vertical transfer of maternally delivered ZIKV with a diverse range of fetal/infant neuropathology. It is less clear if the high rate of vertical transmission of ZIKV in macaques is inherent to these species of primates or is related to the uniquely prolonged maternal viremia in pregnant macaques (routinely a month or longer) that may lead to continued or episodic exposure of the fetus to ZIKV over long periods of gestation, despite the development of neutralizing antibodies.

Despite these elegant studies in pregnant macaques, there is still a clear need to develop additional NHP models to study pregnancy and fetal outcome from ZIKV infection, in particular addressing the early events of vertical transfer. In the present study, we developed the olive baboon (*Papio anubis*) as an alternative NHP model to study ZIKV infection and pathogenicity during pregnancy that can be compared/contrasted with ZIKV infection in human and other NHP pregnancies. Unlike the studies in macaques that focused on late gestation fetal or infant neuropathological outcome, our focus was on the timing of transplacental ZIKV passage and the early mechanistic events of ZIKV induced pathogenesis of the fetal brain. The olive baboon is similar to humans in terms of size, genetics, reproduction, brain development and immune repertoire which makes the baboon an excellent translational NHP model to study ZIKV infection and for vaccine and therapeutics development [41–43]. The baboon has been used as a NHP model for assessing safety and efficacy of vaccines in adults, pregnant females and their infants [42, 44]. The baboon is permissive to flavivirus infection and replication, including ZIKV, and produces a virus-specific immune response [25, 43]. Herein, we describe infection of four timed-pregnant olive baboons at mid-gestation with a contemporary French Polynesian strain of ZIKV (H/PF/2013). The French Polynesian ZIKV strain contains a single point mutation in the prM protein that dramatically increases ZIKV infectivity in both human and mouse NPCs compared to the ancestral African/Asian ZIKV strains [45]. This mutation was conserved during the ZIKV spread through the Americas and is associated with adverse fetal outcomes, including increased microcephaly in French Polynesia [46–49]. We report that the pregnant olive baboon is susceptible to ZIKV infection during gestation including vertical transfer of virus to the fetus resulting in both fetal death as well as fetal cerebral cortical pathologies.

## Results

### Symptomology and pregnancy outcome

All ZIKV infected dams had minor weight loss during the study period (Dam 1: 16.4 start, 16.2 kg end [7 dpi]; Dam 2 16.0 start, 15.6 kg end [14 dpi]; Dam 3: 21 start, 20.9 kg end; 14 dpi; Dam 4: 13.8 start, 13.6 kg end [21 dpi]), however, none of the dams exhibited inappetence. Dam 1 exhibited a mild rash on day three post-infection in the axillary and inguinal regions as well as conjunctivitis that cleared by day seven post-infection; Dam 2 exhibited a mild rash in the axillary and inguinal regions and minor conjunctivitis by day three post-infection which expanded to moderate to severe maculopapular rash on the abdomen and inguinal regions with mild rash on the chest and back of both arms with mild conjunctivitis by day seven that resolved by day 14 post-infection. Dam 3 developed a mild rash in the axillary region and a moderate rash in the inguinal region with moderate conjunctivitis that resolved by day seven post-infection. Dam 4 exhibited a mild rash on day three post-infection in the axillary and inguinal regions as well as mild conjunctivitis that progressed to a mild to moderate rash by day seven-post infection that included the abdomen, chest and backs of arms that resolved by 14 dpi. Body temperatures obtained under ketamine sedation did not show any fever greater than 1°C above day 0 over the course of the study for each animal.

Normal fetal heart rates were obtained for Dams 1, 3 and 4 from the day of inoculation through pregnancy termination on days 7, 14 and 21 post-infection respectively. Dam 2 exhibited normal fetal heart rate through day 7 post-infection. However, on day 14, no fetal heart rate was found and upon subsequent necropsy, fetal demise likely occurred within the preceding 24 hours. There was rupture of the fetal membranes in this pregnancy and signs of meconium staining.

### Maternal ZIKV viral loads

**Fig 1:**
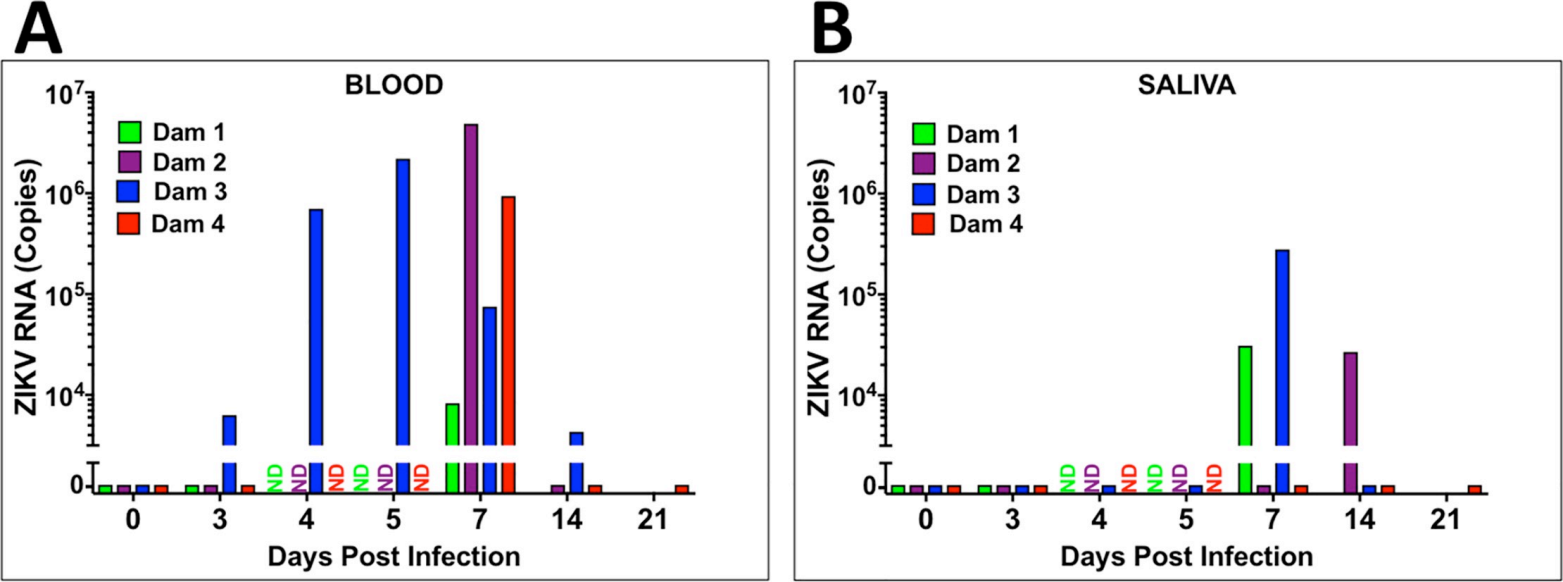
Viral loads (RNA) in whole blood (A) and saliva (B) from ZIKV-infected pregnant baboons. A one-step qRT-PCR was used to measure ZIKV RNA in whole blood (A) and saliva (B) from each animal at indicated days post-infection (copies per milliliter; ND: not determined).

For Dams 1, 2 and 4, ZIKV RNA was not detected in whole blood on day three but was detected on day seven post-infection. In Dams 2 and 4, viremia was resolved by day 14 post-infection (Fig 1A). In Dam 3, ZIKV RNA was detected on days 3 through 14 post-infection (study termination, Fig 1A), albeit the peak viremia was observed at 5 dpi in this dam and had declined by ~3 orders of magnitude by day 14 post-infection. ZIKV RNA was detected in saliva from Dams 1 and 3 at day seven post-infection, and from Dam 2 on day 14 post-infection (Fig 1B).

ZIKV RNA in urine was only detected in Dam 2 on day 14 post-infection (day of necropsy) (urine was not collected from Dam 3; Table 1). Only Dam 4 had ZIKV RNA in CSF (day 7 post-infection). None of the dams had ZIKV RNA in vaginal swabs at any time point. Reproductive tissues (cervix, uterus, ovaries), cerebral cortex and cerebellum were examined for ZIKV RNA from the dams from tissues taken at the time of necropsy. ZIKV RNA was only detected in the uterus of Dam 2 (14 dpi).

**Table 1:** ZIKV RNA in select maternal, placental, fetal tissues (copies per mg tissue or per ml of fluid).

Fluids/Tissues	Dam 1	Dam 2	Dam 3	Dam 4	Dam 5 (Control)
Gestation day start/end	102/109 dG	107/121 dG	101/115 dG	97/118 dG	116/116 dG
Termination post infection	7	14	14	21	-
**Maternal Samples**					
Whole Blood	10^3 (D7)	10^6 (D7)	10^3 (D3)	10^5 (D7)	-
			10^5 (D4)		-
			10^6 (D5)		-
			10^4 (D7)		-
			10^4 (D14)		-
Saliva	10^4 (D7)	10^4 (D14)	10^5 (D7)	<LD	-
Urine	<LD	10^4 (D14)	N/A	<LD	-
CSF	<LD	<LD	N/A	10^3	-
Vaginal Swab	<LD	<LD	<LD	<LD	-
Amniotic Fluid	<LD	10^5	<LD	10^5	-
Uterus	<LD	10^3	<LD	<LD	-
Cervix	<LD	<LD	<LD	<LD	-
**Fetal Samples**					
Fetal Death	No	Yes	No	No	No
Cord Blood	<LD	<LD	<LD	<LD	-
Placenta	<LD (site 1)	<LD (site 1)	<LD (site 1)	10^4 (site 1)	-
	<LD (site 2)	10^6 (site 2)	<LD (site 2)	<LD (site 2)	
	<LD (site 3)	10^4 (site 3)	10^4 (site 3)	10^4 (site 3)	-
	<LD (site 4)	<LD (site 4)	<LD (site 4)	10^4 (site 4)	-
Umbilical cord	<LD	<LD	<LD	<LD	-
Cortex	<LD	10^4 (Parietal)	<LD	10^4 (Frontal)	-
Cerebellum	<LD	<LD	<LD	<LD	-
Lung	<LD	10^6	<LD	10^7	-
Liver	<LD	<LD	<LD	<LD	-
Spleen	<LD	10^5	<LD	10^5	-
Gonad	<LD	10^7	<LD	10^4	-
Stomach	<LD	<LD	<LD	<LD	-
Intestine	<LD	<LD	<LD	10^4	-

<LD (limit of detection); Site 1, 2, 3, 4 represent different placental cotyledons.

Frontal, parietal represent tissues from cortical lobes

### Placental, amniotic fluid and fetal ZIKV viral loads

Fetuses are coded to match the dams (eg. Dam 1 = Fetus 1). We did not detect ZIKV in cord blood obtained at necropsy from any of the four fetuses. Fetus 2 (14 dpi) had ZIKV RNA in placenta, cerebral cortex, lung, spleen and ovary (Table 1). ZIKV RNA was found in Fetus 4 (21 dpi) in placenta, cerebral cortex, lung, spleen, intestine and ovary. ZIKV RNA was detected in amniotic fluid for both Fetus 2 and 4 (Table 1). Fetus 3 (14 dpi) had ZIKV RNA in the placenta and Fetus 1 (7 dpi)did not have detectable ZIKV in any tissue examined or amniotic fluid.

### ZIKV specific IgM and IgG and neutralization (PRNT)

#### IgM

**Fig 2:**
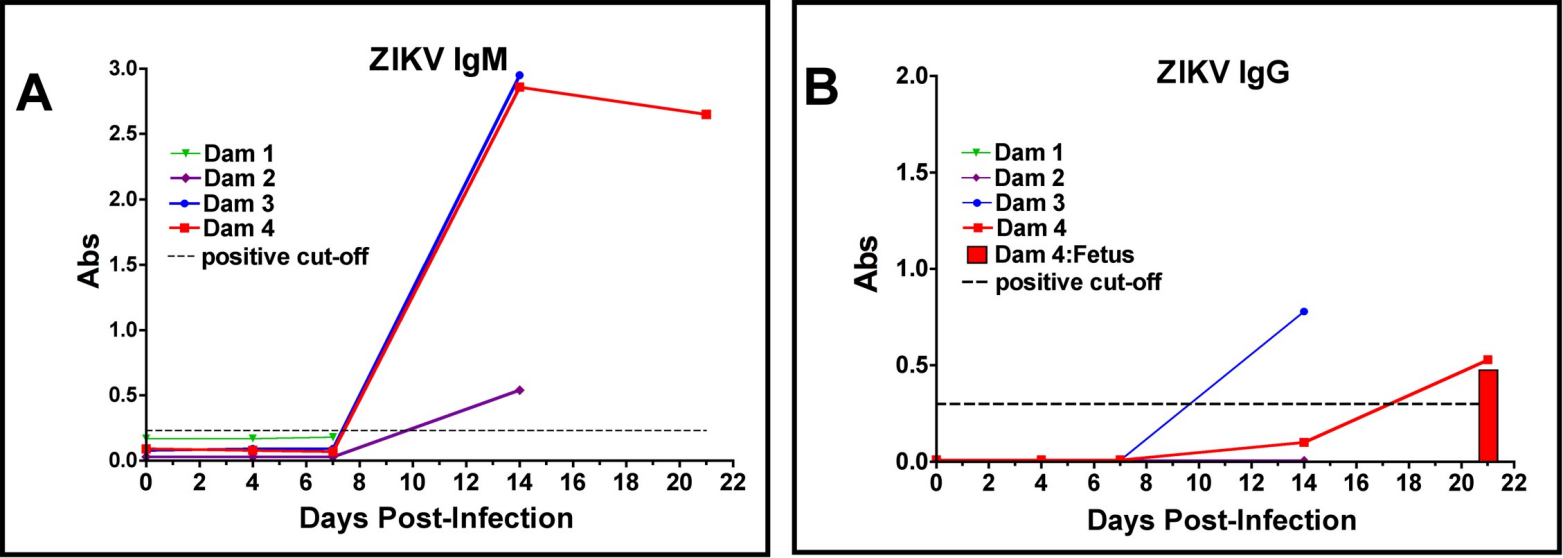
Detection of anti-ZIKV antibody responses in pregnant baboon serum. The presence of antibodies directed against ZIKV was determined by ELISA for IgM (A) or IgG (B). anti-ZIKV IgM were detected at 14 days post-infection in all three baboons sampled at this time point. Only two of the pregnant baboons had anti-ZIKV IgG; one at 14 days and the other by 21 days post-infection. The dam (2) with the weakest IgM response at 14 days did not exhibit an IgG response at this time point. The fetus of Dam 4 had anti-ZIKV IgG at levels similar to the mother indicating efficient FcγR transfer of IgG across the placenta.

All dams were negative for ZIKV IgM prior to infection and remained negative through 7 dpi. The three dams (Dams 2, 3 and 4) sampled at 14 dpi had IgM for ZIKV; Dams 3 and 4 exhibited a robust IgM response, Dam 2 had low ZIKV IgM, ~25% that of the other dams. In Dam 4, ZIKV IgM remained constant from 14 through 21 dpi (Fig 2A).

#### IgG

No dams had serum ZIKV IgG by day seven post-infection. Dam 3 and 4 had ZIKV IgG by 14 dpi and in Dam 4, ZIKV IgG increased from 14 to 21 dpi. ZIKV IgG was also detected in the cord blood of Fetus 4 (21 dpi). Dam 2 did not have ZIKV IgG at 14 dpi; this dam also had low IgM (Fig 2B).

#### PRNT

In the two dams that exhibited an ZIKV IgG response, Dam 3 had a PRNT_50_ of 1:1280 (14 dpi) while Dam 4 had a PRNT_50_ of 1:2560 (21 dpi). The cord plasma from Fetus 4 had a PRNT_50_ of 1:640.

### CNS histology and immunohistochemistry

Upon standard H&E staining, none of the three fetuses from ZIKV infected dams (with available CNS tissue) for histology had gross pathology of the cerebral cortex or other brain structures (Fetus 2 had extensive autolysis of the brain after *in utero* death). Histological examination of the frontal cortex (CNS region with abundant ZIKV RNA; 1x10^4^ copies/mg) of Fetus 4, which exhibited vertical transfer of virus at 21 dpi, revealed no major gross pathological lesions, calcifications, signs of vascular collapse or vasculitis or decreased cortical volume compared to the control fetus or the two fetuses collected at days 7 and 14 post-infection with no evidence of vertical transfer of ZIKV.

### Glial Fibrillary Acidic Protein (GFAP)

**Fig 3:**
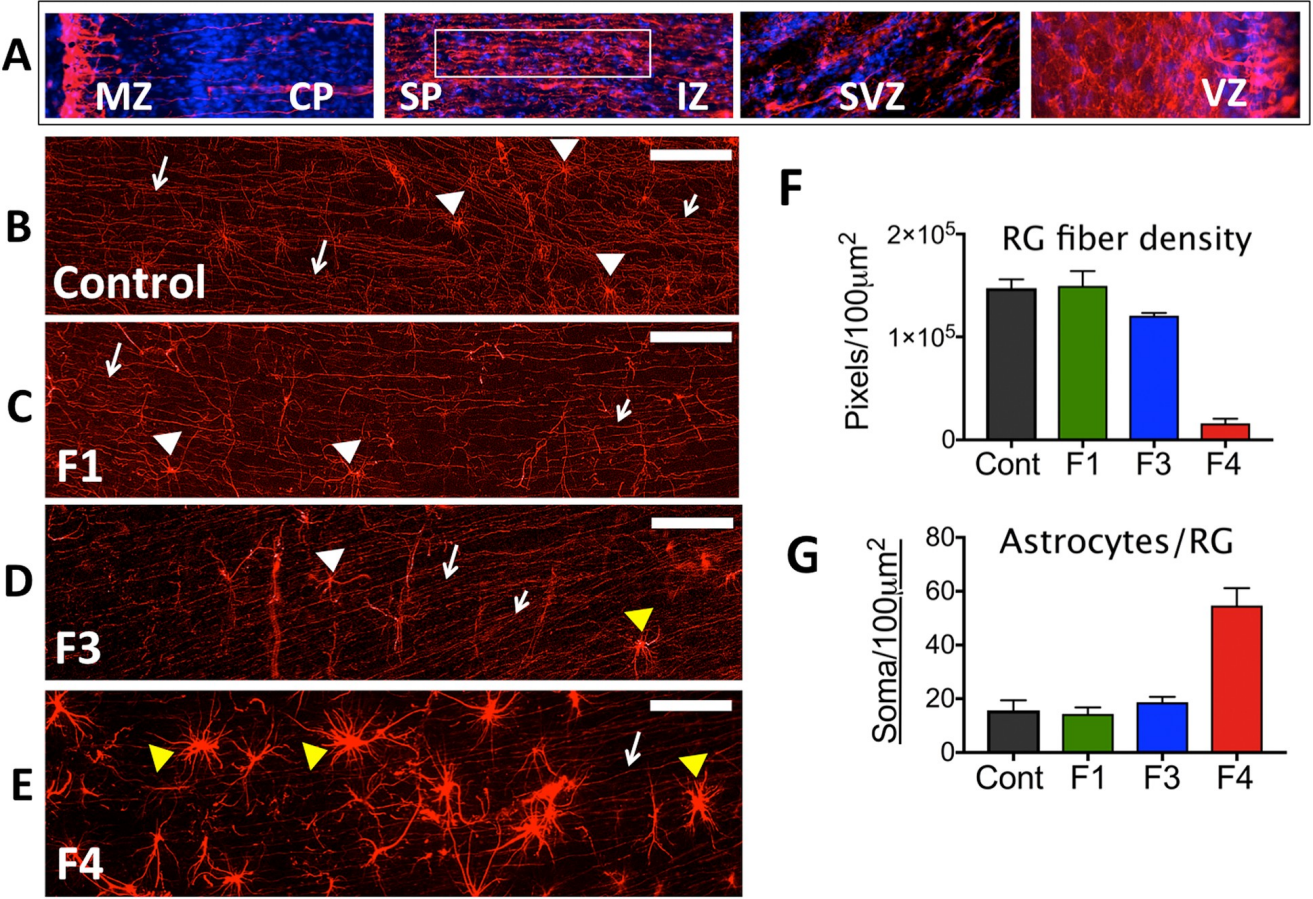
Immunofluorescence (IF) for GFAP in the frontal cortex of fetal baboons from an uninfected control dam and from dams infected with ZIKV. (A) Reconstruction of GFAP-IF in the frontal cortex of the fetal baboon (marginal zone (MZ), cortical plate (CP) subplate (SP), intermediate zone (IZ), subventricular zone (SVZ) and ventricular zone (VZ; (116 days gestation). B-E: GFAP-IF (RED) in the SP of the frontal cortex showing radial glial (RG) fibers (arrows) and occasional radial glia (white arrowheads) or astrocyte-appearing soma (yellow arrowheads) in control (B), and in three fetuses after maternal infection with ZIKV (C-E). Fetus 1 (C; 7 dpi; 109 days gestation) and Fetus 3 (D; 14 dpi; 115 days gestation) had an identical pattern of GFAP-IF RG fibers comparable to the control fetus. Neither Fetus 1 nor 3 had detectable ZIKV RNA in any fetal tissue including any of the cortical lobes. Fetus 4 (E; 21 dpi; 118 days gestation) that had detectable ZIKV RNA and protein in fetal frontal cortex had few continuous RG fibers (arrows). In the control fetus and Fetus 1, 2 and 3, occasional RG soma were noted (white arrowheads) with typically small soma and numerous radiating processes; In Fetus 3, an occasional larger soma consistent with astrocytes (yellow arrowheads) was noted. In Fetus 4, in addition to reduction in RG fibers, a notable dense astrocyte population was observed (yellow arrowheads). (F) Image analysis of astrocyte/radial glia soma (soma/100 μm^2^ of SP imaged per section, average ± SEM for three sections, 150 μm between sections) for each fetal frontal cortex. There was an ~3-fold increase in astrocytes/soma per unit area in the SP of Fetus 4 (F4) compared to the control fetus (Cont) or Fetus 1 (F1) or Fetus 3 (F3). (G) Image analysis of RG fiber density in the SP of each area imaged (GFAP^+^ fibers/100 μm^2^ of SP; average ± SEM for three sections per fetus) demonstrated that Fetus 4 (F4) had less than 10% of RG fibers remaining compared to the control fetus (Cont) or Fetus 1 or 3 (F1, F3). (bar = 25 μm).

Immunofluorescence (IF) for GFAP, a classical marker for radial glia (RG) and astrocytes in the developing cortex, revealed a pronounced difference in the ZIKV infected frontal cortex compared to the control (or day 7 or 14 post-infection fetuses without vertical transfer of virus). In the control fetus and fetuses with uninfected brains (Fetus 1, 3), the anticipated pattern of dense glial fibers projecting from the ventricular zone (VZ) to the marginal zone (MZ) was observed (Fig 3B–3E). However, in the frontal cortex from the ZIKV infected fetus, there was a pronounced decrease in GFAP^+^ fibers, in particular in the subplate (SP) and intermediate zone (IZ; Fig 3F). Image analysis demonstrated that Fetus 4 had ~10% of the RG fibers in the SP/IZ (Fig 3F) compared to the control fetus and the two fetuses from ZIKV infected dams that did not have detectable ZIKV in the brains or other fetal tissues (Fetus1, 3). Concurrent with the loss of RG fibers, a noted increase in the density in astrocytes was observed in the IZ/SP regions of Fetus 4 (~5-fold increase; Fig 3G) compared to the cortex of the control fetus and the two fetuses from infected mothers not exhibiting vertical ZIKV transfer. In the uninfected fetal frontal cortices, GFAP-IF revealed a pattern of sporadic RG/astrocytes with few astral branches representing maturing astrocytes with normal growing processes typical of this gestational age (Fig 3B–3D).

### NeuN and Nestin

**Fig 4:**
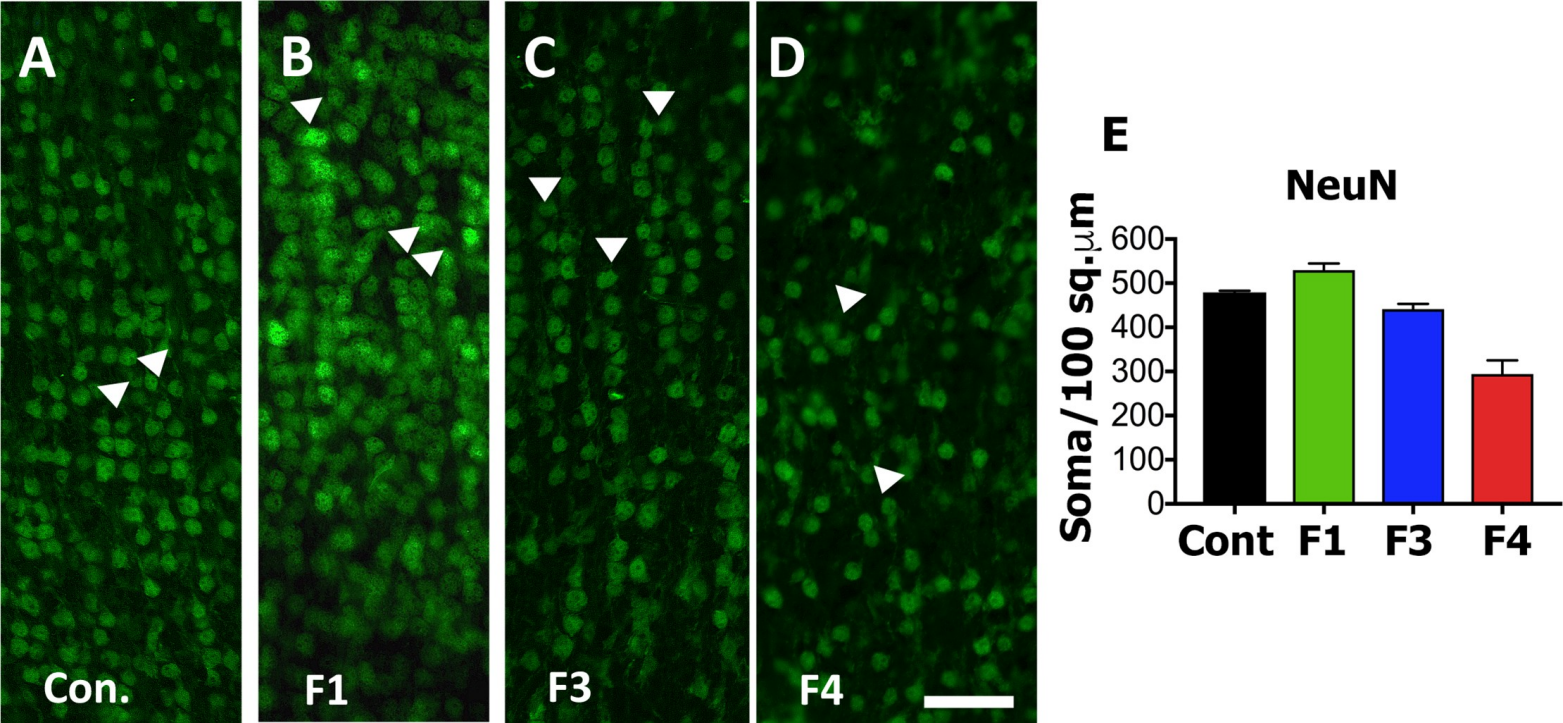
NeuN immunofluorescence (Green) in the cortical plate of the frontal cortex of fetal baboons from an uninfected control dam (A), and from dams infected with ZIKV (B-D). Arrowheads indicate columns of NeuN labeled neurons in the CP of the control fetal cortex (A) and in the CP of Fetus 1 (B) and 3 (C) from ZIKV infected dams without viral transfer to the fetus. In contrast, Fetus 4 (D), which had detectable ZIKV RNA in the frontal cortex at 21 dpi had NeuN IF pattern that showed considerable disorganization consistent with the loss of radial glial fibers in the cortex of this fetus. Image analysis of NeuN+ neurons (E; NeuN^+^ cells/200 μm^2^ for three sections, mean ± SEM; 150 μm between sections) for each fetal frontal cortex (Control: Cont, Fetus 1: F1; Fetus 3: F3; Fetus 4: F4). There was a 2-fold decrease in NeuN^+^ IF neurons in the CP of the ZIKV infected cortex (Fetus 4) compared to the other fetal cortices. (bar = 25 μm).

**Fig 5:**
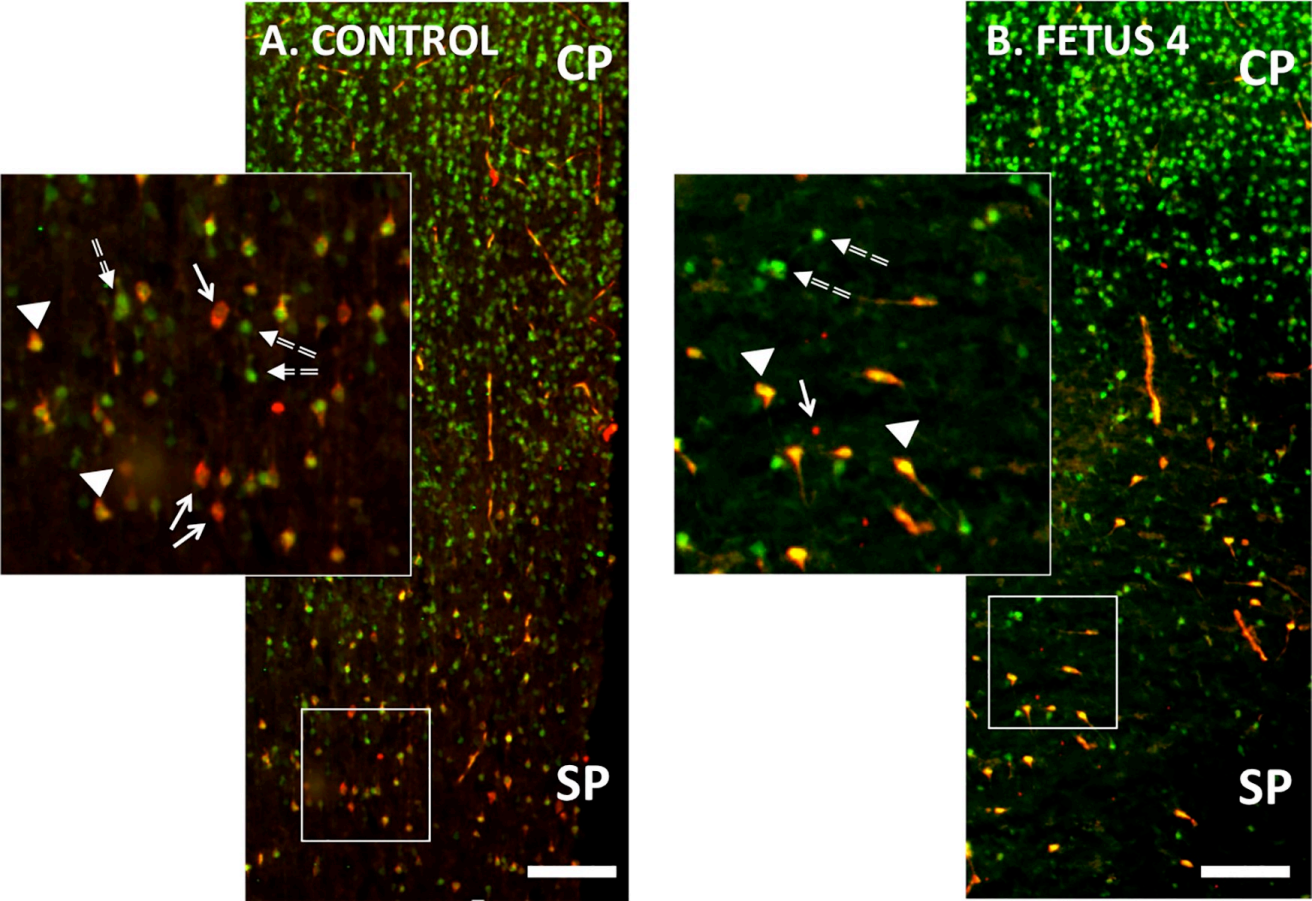
Double immunofluorescence for NeuN (green; differentiating neurons) and Nestin (red; neuroprogenitors; NPCs) in the cortical plate (CP) and subplate (SP) of the developing frontal cortex of the control (A) and 21 day post ZIKV infection (B) fetal frontal cortex (Fetus 4). There were numerous differentiating neurons co-expressing NeuN and Nestin in the control cortex (arrows; yellow) as well as NPCs (Nestin: Red; arrowheads) and differentiated neurons (NeuN: Green; dashed arrows). In the ZIKV infected Fetus 4 (B), Nestin^+^ neurons (Green) appeared disorganized compared to the control cortex (which showed the characteristic pattern of columns of Nestin^+^ neurons migrating from the SP to the CP); only a few scattered remaining NPCs (NeuN: red) or differentiating neurons co-expressing NeuN and Nestin are observed in the ZIKV infected cortex. (bar = 100 μm).

In order to determine if ZIKV infection targeted NPCs or reduced cortical neurons we performed IF for NeuN (neurons, differentiating neurons) and Nestin (NPCs). In the control fetal cortex and the cortices of Fetus 1 and 3 (no detection of ZIKV in the fetus), NeuN^+^ IF positive neurons were observed in long organized tracks of migration in the IZ/SP through the CP, while in the ZIKV infected cortex (Fetus 4), the pattern of NeuN^+^ neurons appeared disorganized and not in the characteristic tracks, even in the CP (Figs 4 and 5). In addition, the number of NeuN^+^ neurons in the CP of Fetus 4 were approximately 50–60% of those observed in the control fetus and the two fetuses from ZIKV infected dams not exhibiting viral RNA in the fetus (Fig 4), indicating that the neuronal migration to the CP had been disrupted after loss of the RG fibers.

Immunofluorescence for Nestin revealed a different pattern in the frontal cortex of Fetus 4 compared to control or Fetus 1 and 3, in particular in the IZ/SP (Fig 5). When observed in the 21 day ZIKV positive cortex, Nestin^+^ IF cells were typically clustered, however there were regions within the IZ/SP that had fewer or were devoid of Nestin^+^ cells in the ZIKV infected Fetus 4. Overall, there appeared to be similar numbers of Nestin+ NPCs in the IZ/SP of Fetus 4, however their distribution was highly altered, again possibly related to the loss of RG fibers noted above.

### O1 (Immature Oligodendrocytes)

**Fig 6:**
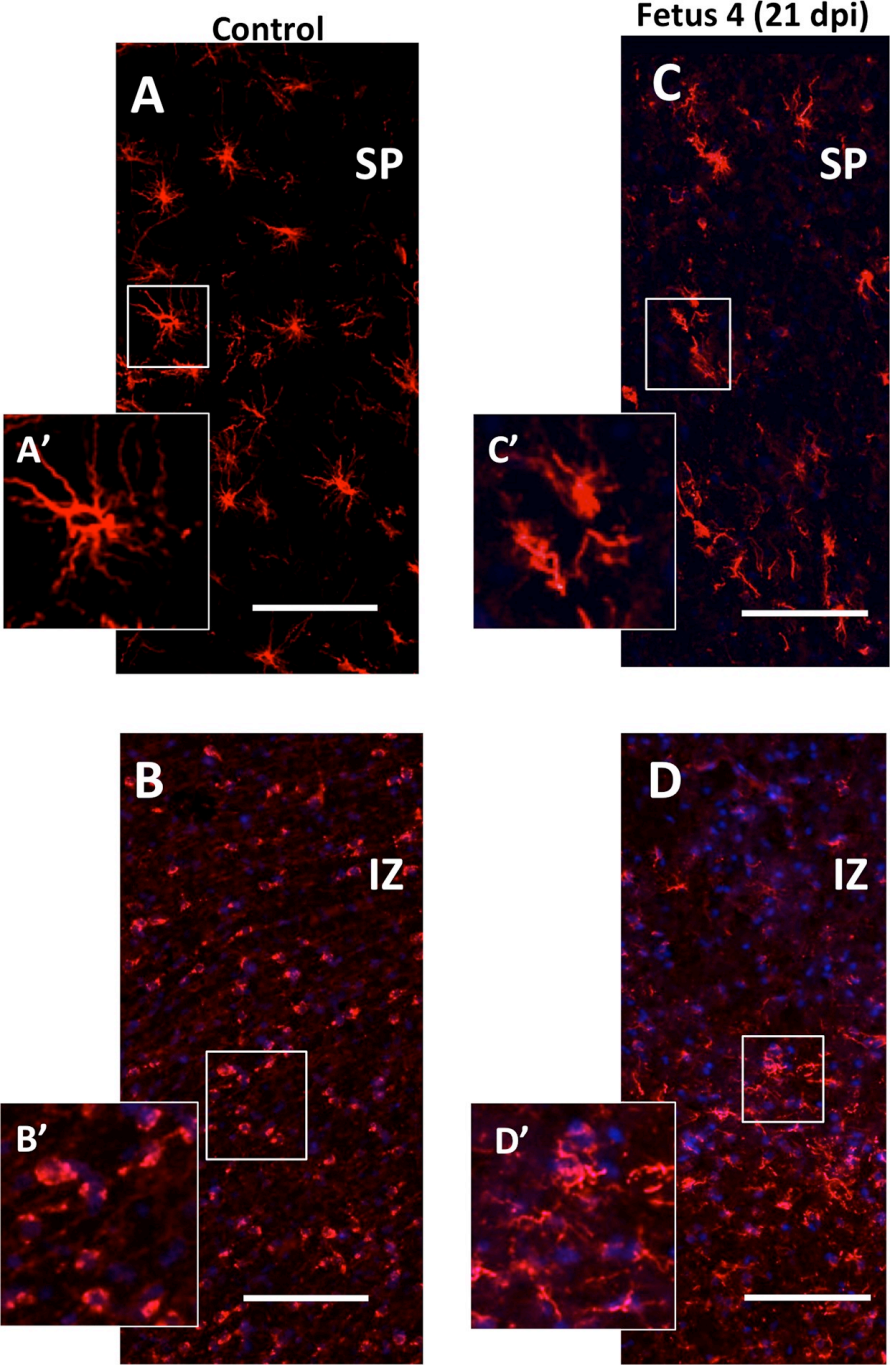
Immunoflurorescence for O1 (immature oligodendrocytes; OL; Red) in the SP/IZ region of the developing fetal cortex. In the control fetal frontal cortex SP (A: SP; B:IZ) there were numerous well developed immature oligodendrocytes (O1+; red) with extensive processes (see inset [A’]) while in the IZ region, the immature oligodendrocytes were smaller, had few processes and more abundant (B; B’: inset showing smaller, less differentiated oligodendrocytes). In the ZIKV infected Fetus 4 (C,D), the number of O1+ cells in the SP (C) and IZ (D) were similar but morphologically distinct with limited processes in the SP and in both SP and IZ, the immature oligodendrocytes had the appearance of degeneration or arrested development. (bar = 50 μm).

In order to determine if ZIKV infection causes white matter damage in term pregnancies and postnatally as reported in ZIKV^+^ fetuses/infants in human population [5] and observed in the 3^rd^ trimester pigtail macaque fetus with ZIKV positive brain [37, 38], we performed IF for O1, a marker for immature oligodendrocytes and the only cell population that matures to oligodendrocytes that are responsible for myelinating the axons. O1 IF showed abundant O1^+^ immature oligodendrocytes in the SP of the control fetal brain (and the cortices of the fetuses without vertical transfer of ZIKV) that exhibited numerous processes typical of immature oligodendrocytes (Fig 6). In Fetus 4, although the numbers of immature OLs were similar in the SP compared to the control fetus, the immature oligodendrocytes were largely without multiple processes and were not evenly distributed as observed for the control fetus (Fig 6). In the IZ, smaller immature oligodendroctyes were observed in the control fetus; these immature oligodendrocytes were largely without processes in the IZ and numerous. In the IZ of Fetus 4, O1 staining revealed that the immature oligodendrocytes were located in the deeper region of the IZ indicating possible disruption of migration and appeared to be in the process of degeneration (Fig 6). The cerebral cortices of Fetus 1 (7dpi) and Fetus 3 (14dpi) appeared similar to the control brain.

### Neuroinflammation

**Fig 7:**
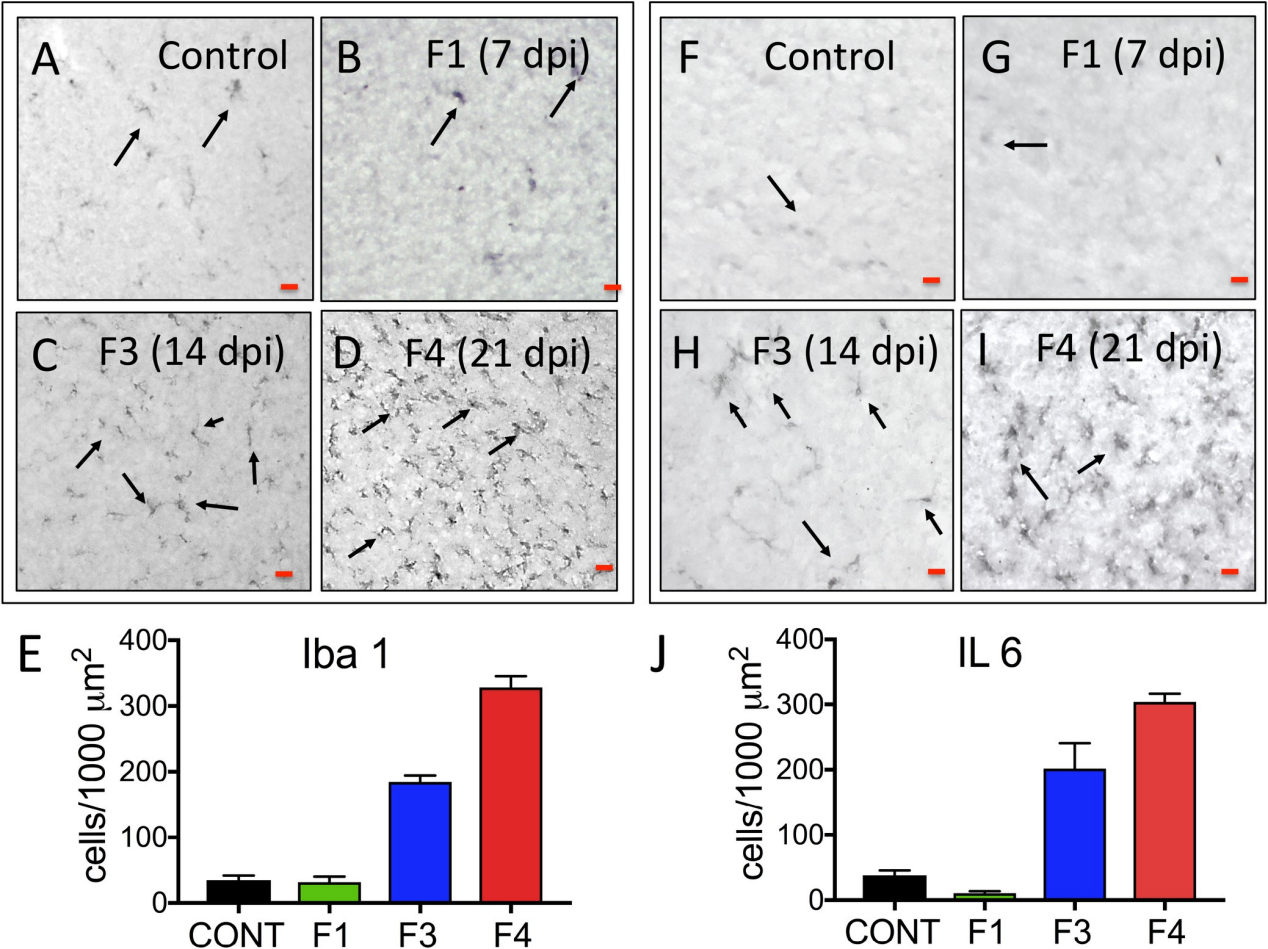
Immunohistochemistry for neuroinflammatory markers (microglia: Iba1; A-D; Interleukin 6 [IL-6]; F-I) in the frontal cortex of control and fetuses from dams infected with ZIKV. In both control (A,F) and Fetus 1 (7 dpi; B,G), occasional Iba1 reactive microglia and IL-6^+^ cells were observed (arrows). In the 14 dpi Fetus 3 (C,H) an increase in the number of Iba1^+^ microglia and IL-6 immunostaining cells were observed throughout the IZ through the CP (E), despite the fetal cortex being negative for ZIKV RNA. The 21 dpi Fetus 4 (D,I) that was ZIKV RNA+, exhibited the greatest Iba1+ microglia and IL-6 immunostaining (J). (For E, J: IF+ cells/200 μm^2^; mean ± SEM for three sections per fetus; 150 μm between sections).

The 21 day ZIKV infected cortex (Fetus 4) exhibited increased neuroinflammation with an approximate 7-fold increase in Iba1 immunoreactive microglia (Fig 7A–7E) and IL-6 (Fig 7E–7H) immunoreactive cells (proinflammatory cytokine) compared to the control fetus or Fetus 1 (7 dpi, no vertical ZIVK transfer). Fetus 3 (14 dpi) had an approximate 4-fold increase in both Iba1 and IL-6 immunostaining in the frontal cortex. While not detecting ZIKV in tissues (including cortex) in this fetus, there was ZIKV RNA and protein in the placenta of this pregnancy. Neuroinflammation was not observed in the day seven post-inoculation frontal cortex (Fetus 1). This fetus did not have ZIKV RNA detected in any fetal tissue or the placenta.

### Apoptosis (TUNEL)

**Fig 8:**
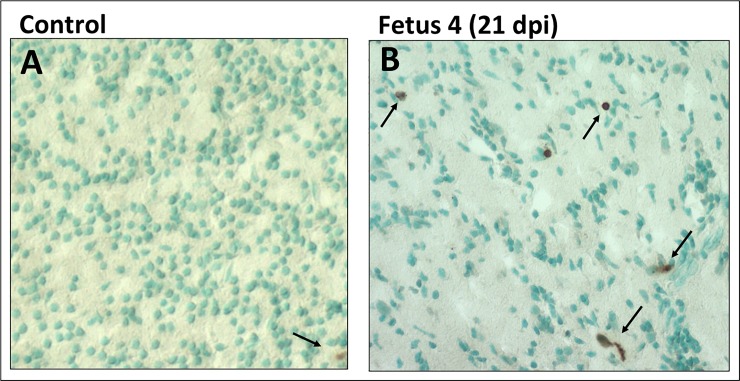
TUNEL staining for apoptosis in the frontal cortex of the control (A) and 21 day post-infection Fetus 4 (B). The cortex of the control fetus had very few apoptotic cells compared to the ZIKV infected fetus (brown cells; arrows). While there were dispersed TUNEL stained cells in the infected fetus, the density was not consistent with widespread apoptosis in the cortex of the infected fetus at 21 days post-infection.

There were little to no apoptotic cells in the control frontal cortex in any region of the developing cortex (Fig 8). There were notable apoptotic cells in the cortex in the day 21 post-infection cortex compared to the control cortex, primarily in the IZ/SP region (Fig 8). The cortex of Fetus 1 was similar to the Control fetus with few apoptotic cells, while the day 14 post-infection fetus exhibited a similar amount of TUNEL staining compared to the ZIKV infected 21 day post-infection fetus.

### Zika virus

**Fig 9:**
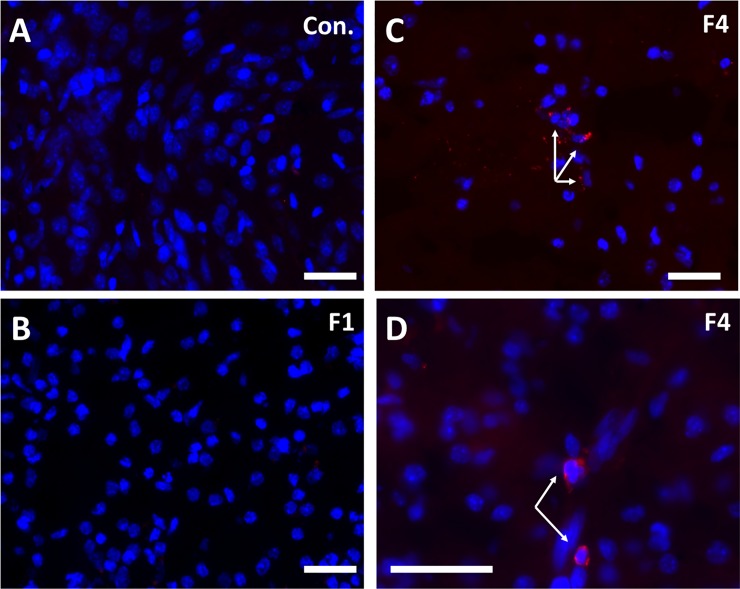
Pan flavivirus immunofluorescence (IF; Red: flavivirus; Blue: DAPI) staining in subplate/intermediate zone in the control fetus (A), 7 day post-ZIKV infection Fetus 1 (B) with no detection of virus in fetal tissues (B) and Fetus 4 at 21 days post-ZIKV infection that was ZIKV RNA+ in the frontal cortex (C,D). There was no IF for ZIKV in frontal cortex in either control (A) or 7 dpi fetus (B). Scattered ZIKV+ IF (Red) was detected in the SP/IZ of fetus 4 that was primarily perinuclear in localization (C 10x, D, 20x objective magnification; bar = 25μm).

Immunofluorescence for ZIKV (pan-flavivirus) revealed focal presence of ZIKV in the frontal cortex of Fetus 4 but not in Fetus 1, 3 or the control fetus, confirming localization of ZIKV in Fetus 4 with high vRNA burden in the frontal cortex. The viral IF was most notable in the subventricular zone (SVZ) and intermediate zone (IZ) (Fig 9).

### Placental histology

Routine H&E staining showed only minor evidence of inflammation in Dam 1, while the placenta of Dam 3 was histologically similar to the control placenta, despite having one cotyledon positive for ZIKV RNA. Similarly, Dam 4, which had vertical transfer of ZIKV to the placenta and fetus, exhibited only minor indices of inflammation. The placenta of Dam 2 (intrauterine fetal death) exhibited significant placental pathology, with extensive fibrin deposition in the intervillous space with nearly uniform degenerated villi with frequent necrosis and acute inflammation.

### Zika virus immunofluorescence

**Fig 10:**
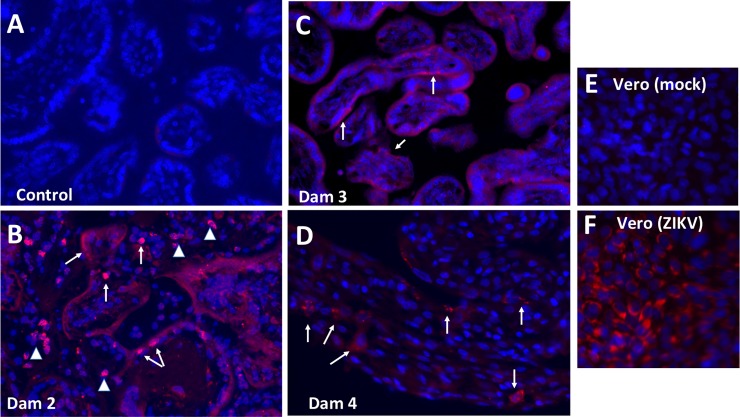
Pan flavivirus immunofluorescence (IF; Red: flavivirus; Blue: DAPI) staining in placenta from control (A), and three placenta from ZIKV infected dams that ZIKV RNA+ in placenta (B, Dam 3, 14 dpi; C, Dam 3, 14 dpi; D, Dam 4, 21 dpi). The most intense IF was observed in the placenta of Dam 2 (B) in which there was fetal death. ZIKV IF was observed in syncytiotrophoblast (arrows) in Dam 2 and 3. Arrowheads in B indicate IF in the villous cores which could be either stromal or endothelial cells. A less intense IF pattern was observed in Dam 4 (D) in the overlying syncytiotrophoblast which is covering the underlying cytotrophoblasts, despite widespread vertical transfer of ZIKV to the fetus.

The control Dam and Dam 1 (7 dpi) were negative for ZIKV IF. ZIKV IF (pan flavivirus) in the placentas of Dams 2 and 3 (14 dpi; both positive for ZIKV RNA) demonstrated the presence of ZIKV, localized primarily in the syncytial layer with regions exhibiting greater intensity (Fig 10). Dam 2, which had fetal demise and ZIKV RNA detected in fetal tissues, exhibited the most intense ZIKV IF and also had IF signal in villous cores, in either stromal or enodothelial cells. Dam 4 (Fig 10; 21 dpi; placenta and fetus ZIKV RNA positive) also exhibited ZIKV IF in the syncytial layer, although the signal was not as widespread as observed in the 14 dpi placentas potentially indicating a decrease in viral replication in the placenta by 21 dpi.

### Plasma cytokine and chemokine response to ZIKV infection

**Fig 11:**
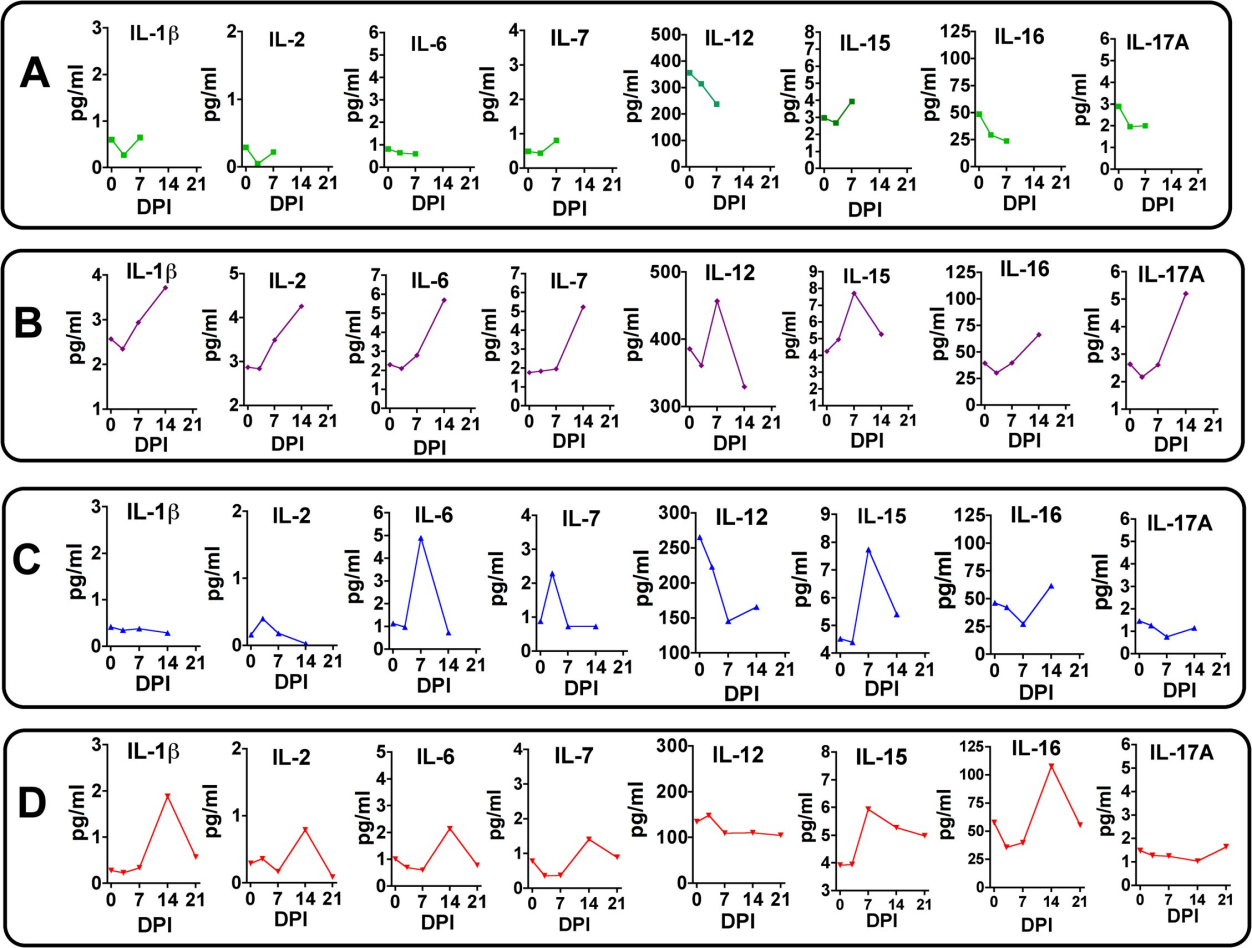
Maternal plasma cytokine concentrations in response to ZIKV infection in timed pregnant baboons. Only cytokines that were detectable and exhibited changes in concentration in response to ZIKV infection are shown. In Dam 1 (A) studied for 7 days post-infection, no chemokines increased post-infection. For Dam 2 (B), increases in IL-β, IL-2, IL-7, IL-12, IL-15, IL-16 and IL-17A were observed with peak levels either at day 7 or 14 post-infection. For Dam 3 (C) acute (day 7) increases were observed for IL-6, IL-7 and IL-15 (with a small increase at day 7 for IL-2). For Dam 4 (D) increases in IL-1β, IL-2, IL-6 IL-7, IL-15 and IL-16 were observed with peak levels either at day 14 (exception IL-15 at day 14), with resolution by study termination at day 21 for this dam.

The cytokine/chemokine response was highly variable between dams. Dam 1 was notable in that no discernable change in any cytokine/chemokine was observed on day three or seven post-infection (study termination, Fig 11A). Dam 2 had an increase in IL-1β, IL-2, IL-6, IL-7, IL-12, IL-15, IL-16 and IL-17A, peaking on day 14 post-infection for all but IL-12 and IL-15 which peaked on day 7 post-infection (Fig 11B). Dam 3 exhibited an increase above baseline in IL-2, IL-6, IL-7, and IL-15; notably, all cytokines increased on day 7 and returned to basal by day 14 post-infection (Fig 11C). Dam 4 exhibited an increase above baseline in IL-1β, IL-2, IL-6, IL-7, IL-15 and IL-16 (Fig 11D), and similar to Dam 2, peak levels of these cytokines were on day 14 post-infection with the exception of IL-15 (day 7); by 21 dpi, most cytokines had returned to baseline or were lower than peak levels.

**Fig 12:**
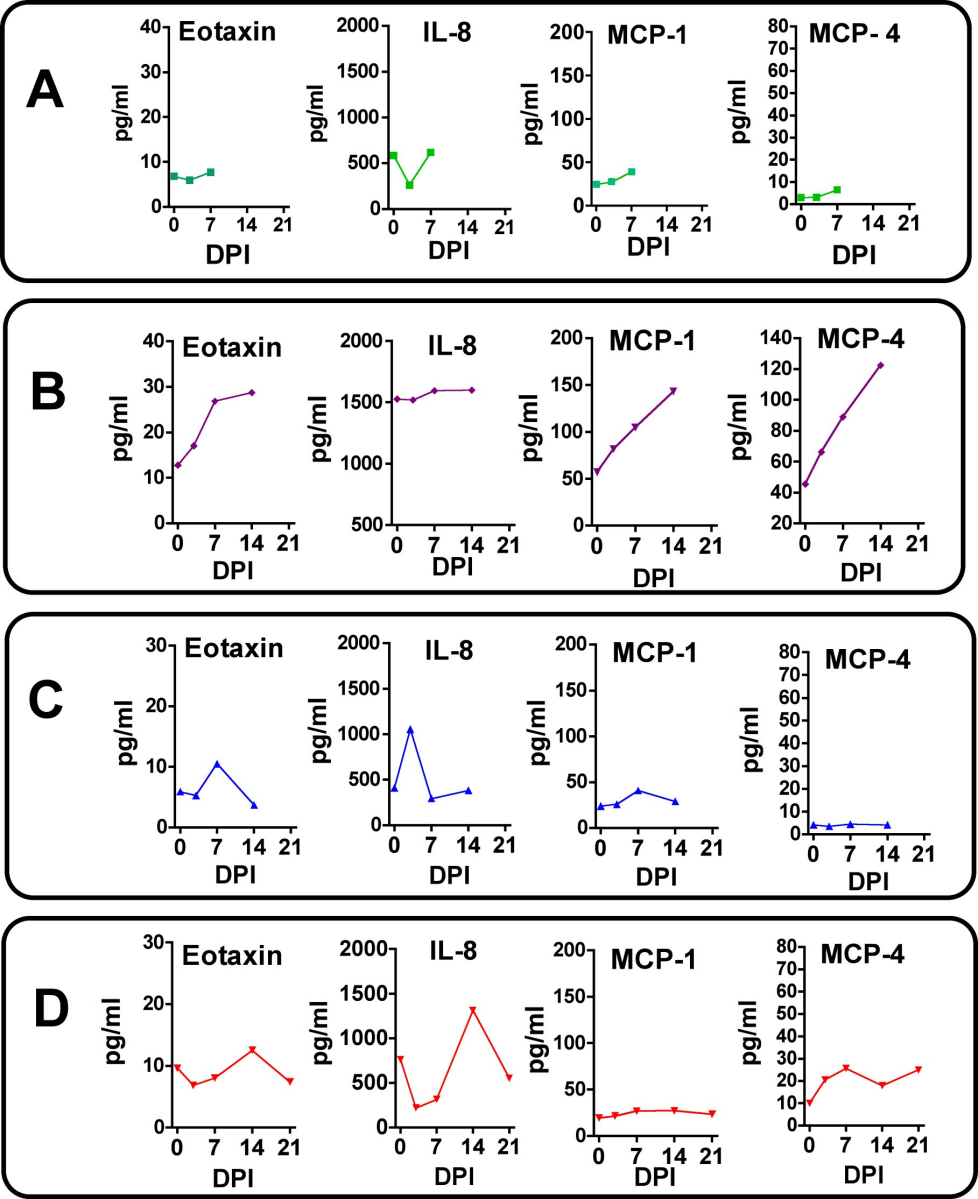
Maternal plasma chemokine concentrations in response to ZIKV infection in timed pregnant baboons. Only chemokines that were detectable and exhibited changes in concentration in response to ZIKV infection are shown. In Dam 1 (A) studied for 7 days post-infection, no chemokines increased post-ZIKV infection. For Dam 2 (B), increases in Eotaxin, MCP-1 and MCP-4 were observed peaking at day 14 post-infection. For Dam 3 (C) a transient increase in Eotaxin and IL-8 was observed. For Dam 4 (D), a similar transient increase in Eotaxin, IL-8 and MCP-4 was noted.

Similar to that seen for cytokines, Dam 1 did not display any notable increase in plasma chemokine levels post-infection (Fig 12A). Dam 2 had notable increases in plasma levels of Eotaxin, MCP-1 and MCP-4 (Fig 12B), and similar to cytokines for this Dam, chemokines exhibited a progressive increase from Day 0 through study termination on Day 14. Dam 3 exhibited small transient increases in Eotaxin and IL-8 on day 7 (Fig 12C). Dam 4 had increases in plasma levels of Eotaxin (small), IL-8 and MCP-4 peaking primarily on day 14 post-infection (Fig 12D).

## Discussion

In this study, we describe ZIKV infection in four olive baboons at mid-gestation (97–107 dG; term ~183 dG) following sc delivery of a relatively modest dose (1x10^4^ ffu) of the French Polynesian isolate resulting in vertical transfer of the virus to the fetus associated with fetal demise in one pregnancy and significant fetal CNS pathology in a second pregnancy. We chose the French Polynesian isolate since the mutation in the prM protein (S139N) of the ancestral Asian ZIKV strain arose prior to the French Polynesian outbreak, and has been stably maintained in the strains circulating in the Americas. This mutation was shown to significantly enhance infectivity in human NPCs and yielded a more significant microcephaly in mice [45]. A retrospective study reported an increase in microcephaly and CZS following the ZIKV epidemic in French Polynesia [49].

Following ZIKV infection, all four pregnant dams exhibited viremia within the first week post-infection and all presented with rash and conjunctivitis varying from mild to moderate, similar to that we described for male and non-pregnant female baboons [25]. Most clinical signs in pregnant humans, including rash, resolve within a week but may last up to two weeks. Description of rash following ZIKV infection in humans has been variable with estimates ranging from relatively infrequent (~1 in 5) [4] to greater than 2/3^rds^ of definite ZIKV cases in a Brazilian pregnancy cohort [50]. As such, the presence and duration of rash and conjunctivitis in our pregnant baboons resemble that observed in human pregnancy. While the magnitude of viremia achieved in the pregnant baboons was similar to that described in our prior study of male and non-pregnant female baboons [25], and pregnant and non-pregnant macaques receiving a similar dose and route of delivery of ZIKV (French Polynesian or other strains), the onset of viremia in three of the four pregnant baboons was slightly delayed (detected at 7 but not 3 dpi) compared to male and non-pregnant female baboons where peak viremia was obtained routinely at 3 to 4 dpi and typically resolved by 7 to 10 dpi. The course of viremia in the pregnant baboons was also different than that described for pregnant macaques, which characteristically show viremia that initiates very early post-inoculation (1–2 days post-inoculation), but is unusual in that it is routinely reported to be prolonged with viral RNA detectable in blood for several weeks up to 70 dpi [16, 31, 34–36]. Viremia was observed in one dam to 14 dpi, albeit declining by >3 orders of magnitude from the peak at 5 dpi, although it is possible that viremia might have been prolonged for this dam if the time frame of her study had been extended. It is noteworthy that this dam also exhibited early viremia, detected at 3 dpi. In humans, viremia following ZIKV infection is usually short-lived (3–7 days) with occasional longer durations of up to 10 to 14 days [4]. While prolonged viremia (46 to 53 days) has been reported in pregnant women [51], it should be noted that this was restricted to five cases after a search of the entire U.S. Zika Pregnancy Registry and as such, prolonged viremia during pregnancy in women appears rare. These authors did not find a correlation between prolonged viremia and an increased incidence of CZS.

Vertical transfer of ZIKV described thus far in macaques appears to be very efficient (100%). We observed vertical transfer to the placenta in three of the four pregnant baboons infected at mid-gestation, two at 14 dpi and the third at 21 dpi. Further vertical transfer to the fetus was observed in two dams, in one dam, vertical transfer of ZIKV was associated with intrauterine fetal death by day 14 post-infection (Dam/Fetus 2), while in a second dam, vertical transfer associated with significant cerebral cortical neuropathology in the fetus at day 21 dpi (Dam/Fetus 4). The latter fetus was otherwise healthy with no congenital anomalies or signs of growth restriction. ZIKV RNA was detected in both fetuses in cerebral cortex, lung, spleen, and ovaries, and additionally in the intestine in Fetus 4 at 21 dpi. Unfortunately, *in utero* fetal death precluded meaningful cortical histopathology of Fetus 2. ZIKV RNA was detected in the amniotic fluid of both of these pregnancies as well. We sampled three to four separate sites (cotyledons) of each placenta since a recent study in macaques indicated that ZIKV infection of the placenta might be localized and not diffuse [32]. In Dam 2 (14 dpi), ZIKV was observed in two cotyledons, in Dam 3, ZIKV RNA was detected in one cotyledon while three cotyledons were positive for ZIKV RNA in Dam 4. Immunofluorescence for ZIKV (pan flavivirus) verified the presence of ZIKV in the placenta of all three dams with ZIKV RNA. Of interest, IF revealed ZIKV in the syncytiotrophoblast layer of all three placentas to varying intensities with the day 14 post infection placenta exhibiting the most uniform IF in the syncytiotrophoblast layer while there was restriction of the IF signal by 21 dpi indicating potential resolution of placental infection by this time post-infection. In situ hybridization for ZIKV RNA has been shown in macaque placental villi, collected at 14 dpi, consistent with our IF findings [34]. Other studies in macaques have focused on long-term infection with collection of the fetus and placenta at late gestation (or delivery) and as such, our findings are of the first to show the early infection of the placenta and targeting of the syncytiotrophoblast by ZIKV. While we did not detect ZIKV RNA in the placenta of Dam 1 (study terminated at 7 dpi), it can be argued that terminating study in this dam at 7 dpi may have precluded placental infection (vertical transfer) and suggests that transfer of the virus to the placenta and fetus occurs at the 2–3 week post-infection period. It is possible that viral escape from the placenta to the fetus and/or amniotic fluid could have been delayed in Dam 3 as well, since we detected ZIKV RNA in the placenta and this dam also had the longest duration of viremia. Based on our observations, vertical transfer in baboons would appear to take place at some point between peak maternal viremia (7 dpi) and 21 dpi, and as such, a relatively early event during ZIKV infection. In support of our findings of a rapid transfer of virus to the fetus, Hirsch et al [32] observed vertical transfer in two late 2^nd^ trimester rhesus monkeys within 20 dpi and vertical transfer with fetal death was noted in an additional study of rhesus macaques at approximately three weeks post-infection [34]. However, this does not preclude vertical transfer from occurring at a later time point post-infection since it has been suggested in pregnant women that vertical transfer may take up to 5 weeks. Similarly, in marmosets, sc infection during early gestation led to vertical transfer with fetal death and miscarriage at 16–18 dpi [39]. In macaques, vertical transfer may also occur over an extended period of time post-infection since the duration of viremia in macaques is unusually prolonged with widespread infection of maternal tissues including immune-privileged sites such as the LN’s and the CNS which could be potential viral reservoirs for future infections. This could provide longer periods for virus to cross the placental barrier as suggested by Nguyen et al [31], since these studies were mostly done in early and mid-gest and taken to term or near-term pregnancy. Clearly, future studies are needed to follow ZIKV infected pregnant baboons for longer periods post-infection. To our knowledge this is the first study in NHPs where the primary focus was on the early post-infection time course of vertical transfer of ZIKV.

Fetal death, miscarriage and preterm birth have been attributed to ZIKV infection in humans [3]. In symptomatic women, a 5.8% miscarriage rate and 1.6% stillbirth was reported for women infected with ZIKV in the first trimester [52]. However, this is likely an underestimate since a majority of ZIKV infections (~60–80%) are asymptomatic [53]. A recent aggregate communication between National Primate Research Centers concluded that fetal death occurred in 26% of ZIKV infected pregnant macaques [40]. Our finding of one case of fetal death in the four infected baboon pregnancies is consistent with that reported for macaques and supports studies in macaques that miscarriage and fetal demise are likely considerably higher in women than currently estimated. In our case, fetal demise occurred at two weeks post-infection similar to the timing of fetal death noted in two macaque studies and in a study in marmosets [34, 35, 39]. It is of interest that the pregnant baboon in the present study with *in utero* fetal death exhibited a potentially delayed or suboptimal immune response to ZIKV with low IgM titers found at day 14 and an absence of IgG titers against ZIKV at day 14 post-infection. This failure to mount an immune response in spite of the highest viremia may have contributed to the rapid vertical transfer and fetal demise. While this dam also exhibited a notable systemic cytokine/chemokine response, it is unclear if this cytokine response played a role in fetal demise or was a result of placental pathology and fetal death. Two other dams studied to 14 and 21 days elicited an IgM response by 14 dpi as well as having IgG titers and robust ZIKV neutralizing capacity by day 14 (Dam 3) and day 21 (Dam 4) post-infection. Again, it is tempting to suggest that the earlier neutralizing IgG response in Dam 3 may have provided some protection to vertical transfer while the delayed IgG response in Dam 4 was insufficient to prevent vertical transfer even though there was efficient transfer of the IgG to the fetus. Unlike the case with fetal demise, Dam 3 exhibited a rather restricted cyto-chemokine response that resolved by day 14. The systemic cytokine response in Dam 4, that also exhibited vertical transfer of virus to the fetus, was delayed, peaking at day 14 post infection and returning to baseline by 21 dpi. Dam 1 was noteworthy in that there was no noted increase in plasma cytokines at either day 3 or 7 (study terminated) despite a robust viremia and rash. While the fold increases in these cytokines is of similar magnitude as that described in humans in response to ZIKV in the acute phase of infection [54], the individual cytokine response to ZIKV infection in pregnant baboons appears quite variable.

Similar to what has been described in macaques, rupture of the fetal membranes was noted in the pregnancy with fetal demise (Dam 2), consistent with ZIKV RNA in both amniotic fluid and urine. It is noteworthy that this is the only one of the four infected dams in which we observed ZIKV RNA in urine, despite detecting the virus in the amniotic fluid of the 21-day post-infection dam (Dam 4), which also had vertical transfer of ZIKV to the fetus. The fetal membranes of that dam were intact and the fetus otherwise appeared healthy with no meconium staining and normal weight for gestational age. An array of placental pathologies ranging from mild to severe have been described in macaques in response to ZIKV infection that includes deciduitis, chorioamnionitis, villitis and calcifications. In the study by Hirsch and colleagues [32], in addition to a range of placental histopathologies, using advanced MRI methods the authors observed indices of placental dysfunction suggesting that ZIKV infection may impact transplacental oxygen transfer resulting in decreased fetal oxygenation. Martinot et al [34] noted the most severe placental outcome in rhesus macaques in response to ZIKV infection that included multiple placental infarctions, chorioamnionitis and villitis. In addition, these authors also noted extensive placental vascular pathologies that included vasculitis, thrombosis and vascular collapse and sclerosis of the villus vessels consistent with severely perturbed fetal oxygenation. With the exception of placental pathologies associated with early fetal death in macaques, the placental pathologies described to date have been at near term or term gestation and the outcome of a ZIKV infected pregnancy (with the noted extended maternal viremia and potential chronic re-exposure of the placenta to ZIKV). Hypoxia is a known fetal stressor and can itself adversely impact fetal CNS function and development depending on the degree of hypoxia. In addition, hypoxia may alter the fetal CNS susceptibility to ZIKV. In the present study on pregnant baboons, only Dam 2, with intrauterine fetal death, exhibited significant placental pathology, with extensive fibrin deposition in the intervillous space with nearly uniform degenerated villi with frequent necrosis and acute inflammation. While we can’t determine if loss of placental function contributed to fetal demise in this dam, placental inflammation is associated with pregnancy loss in women and this Dam exhibited the highest systemic cytokine response. The placentas of two other baboons exhibited minor evidence of inflammation (Dam 1 and 4) while the placenta of Dam 3 was histologically similar to the control placenta, despite having one cotyledon positive for ZIKV RNA. It is possible that we did not histologically evaluate a placental region infected with the virus in this dam. Hirsch and colleagues observed that ZIKV infection in the placenta may be focal rather than diffused with cotyledon by cotyledon variability in presence of ZIKV RNA in their study of rhesus macaques. We noted similar regional variation in ZIKV detection in placentas and as such, placental pathology may reflect restricted sampling of tissue for histology.

A variety of fetal or infant neuropathologies have been described following infection of pregnant macaques with various strains of ZIKV ranging from none to significant [31, 33, 34, 37, 38]. Thus far, the studies in macaques have focused on late gestation fetal or infant neuropathology. We chose to examine the early events of ZIKV infection on the fetal CNS to shed light on the initial events of ZIKV induced CNS pathology in an NHP, since late gestation or infant CNS outcome is likely the cumulative result from ZIKV targeting of cells as well as chronic inflammation and the long-term effects of placental dysfunction (eg. chronic fetal hypoxia, hypoxia/ischemia, and nutrient restriction). While ZIKV RNA was detected in the brains of two fetal baboons (one at 14 dpi; one at 21 dpi), one fetus died *in utero* and the brain was unavailable for pathologic analysis due to necrosis. However, one of the major findings in the present study was the noted neuropathology in fetal frontal cortex at 21 dpi. To our knowledge, this is earliest description of CNS neuropathology post-ZIKV infection in a primate in a viable fetus that was not aborted or underwent *in utero* death. A pronounced difference in immunostaining for GFAP, a marker for Radial Glia (RG), was noted in the ZIKV infected frontal cortex compared to the control fetus (and the two fetuses from ZIKV dams with no detectable vertical transfer of virus to the fetus). In the ZIKV infected frontal cortex (Fetus 4) a substantial loss of radial glial fibers was observed (~90% reduction in density), in particular in the IZ/SP region. Radial glial fibers serve as scaffolding for migrating neurons and neuronal precursors to the cortical plate to form the characteristic six-layered cortical structure [55–59]. To our knowledge, this is the first description of loss of RG fibers in primates in response to ZIKV. In the present study, we observed an ~50% decrease in the number of NeuN neurons in the cortical plate of Fetus 4 compared to the control fetus or the two fetuses without vertical transfer of ZIKV (Fetus 1, 3). In addition, NeuN neurons in the cortical plate of these fetuses were organized into the typical pattern of rows following RG fibers, while in the ZIKV infected frontal cortex, the NeuN neurons were appeared disorganized. Since macaque studies to date have focused on late gestation or neonatal outcomes, it is unknown if a loss of RG fibers also occurred in response to ZIKV in these studies. However, Waldorf-Adams and colleagues [37, 38] noted that neural stem cells in the SVZ of the temporal cortex appeared disorganized in the ZIKV infected pigtail macaque when examined at late gestation. A similar disorganization and/or abnormal migration of NPCs was noted in infant rhesus macaques from dams infected with ZIKV in early or mid-gestation [34]. These studies are suggestive that RG fiber loss possibly occurred in response to ZIKV as well in the macaque.

In addition to serving as neural stem cells and providing scaffolding for neuronal migration in the cortex, RG also differentiate to astrocytes and pre-oligodendrocytes [58–61]. Normal transformation of RG to astrocytes in the human fetal frontal cortex takes place gradually over several weeks, mainly in the second half of gestation [[56]-57]. Concurrent with the loss of the RG fibers, a dramatic increase (~5-fold) in RG/astrocytes was observed in the ZIKV infected frontal cortex in the ZP/IZ (Fetus 4). In the control fetus and the two fetuses lacking vertical transfer of virus, GFAP staining followed the expected pattern of RG/astrocyte distribution. The expanded population of astrocytes in the ZIKV infected cortex suggests a rapid induction of differentiation of RG to astrocytes (away from neuronal differentiation) coupled with potential migration of astrocytes into this region, rather than death of the RG *per se* concomitant. In the prior study in the pigtail macaque, vertical transfer of the Cambodian strain of ZIKV also resulted in an increase in GFAP- stained astrocytes in the white matter of the cortex as observed at six weeks after infection (at near-term gestation) [37]. In mice, ZIKV delivery directly into fetal brains results in extensive microglial activation and astrogliosis, consistent with our findings[11]. These authors noted that the GFAP immunostaining reflected a loss of RG and a progression of protoplasmic astrocytes into reactive astrocytes. The findings of our study support that an early event following ZIKV penetration of the fetal CNS may be accelerated RG differentiation to astrocytes, in particular in the IZ/SP where the RG are primarily located during this stage of development coupled with a loss of RG fibers.

Astrogliosis is a normal response to viral infection and brain injury and our results and that reported for the pigtail macaque are in agreement with this. In pigtail macaques [37, 38], the authors suggested that ZIKV infection induced periventricular white matter injury resulting in the increased white matter gliosis and increased population of astrocytes. In humans, RG differentiate into pre-oligodendrocytes (preOL), [62–64]. Thus, a loss of RG could conceivably reduce subsequent formation of OL and reduce myelination consistent with the observations in the pigtail macaque and human fetuses obtained from ZIKV infected pregnancies [37, 38]. In the control fetus, we observed an abundant population of O1^+^ immature oligodendrocytes exhibiting the characteristic multi-branched projections in a gradient from the IZ/SP through the CP and developing white matter similar to mid-gestation human fetus [65]. In contrast, in the 21 day ZIKV infected fetal cortex, the O1+ cells were primarily without processes, fewer in number with the appearance of undergoing degeneration. In primates, cortical white matter forms within, and eventually replaces the IZ/SP and as such, the effects of ZIKV infection observed in our study may disrupt normal white matter development [61]. Again, these findings are consistent with that reported in the pigtail macaque in which a primary outcome from ZIKV infection later in the second trimester was reduced white matter.

Direct ZIKV infection into fetal mouse brains showed that a predominant outcome was loss of NPCs, either through apoptosis or altered cell-cycle regulation and decreased differentiation [11, 66]. Loss of NPCs or NSCs have been described in the late gestation fetal or infant macaque CNS following ZIKV infection in early or mid-gestation. Waldorf-Adams and co-workers [38] noted a decrease in TBR2+ intermediate precursors in the SGZ of the dentate gyrus at late gestation in the pigtail macaque following either early or mid-gestation infection with ZIKV. While cortical neurons are largely formed early in gestation through mid-gestation in primates, the dentate gyrus maintains a neurogenic niche well past birth to adulthood. However, in this study, the authors reported a loss of non-cortical (white matter) volume and corticogenesis appeared normal, even in fetuses where the dam was infected in early gestation during a period of major cortical neurogenesis and migration. Coffey et al.,[33] found reduced NPCs (Nestin/ Sox2+) in the dentate gyrus of late gestation fetal rhesus who were exposed to a combined simultaneous intra-amniotic and subcutaneous route of infection Interestingly, Martinot et al.,[34] reported an apparent increase in NPCs in the prefrontal cortex, frontal cortex and basal ganglia and increased apoptosis of NPC’s in the SVZ in rhesus monkeys examined at birth that were infected either early or mid-gestation. These authors identified NPCs by standard histological staining and not immunochemical identification. We observed that Nestin^+^ cells in the cortex of Fetus 4, when observed, were typically clustered and appeared disorganized. In our study, the degree of apoptosis (TUNEL staining) does not appear to support a mass targeting of NPCs by ZIKV in the mid-gestation fetal baboon cortex. Considering the decreased number of NeuN neurons in the cortical plate, coupled with large regions of SP/IZ with sparse populations of Nestin^+^ cells, apoptosis may have been a transient, early event during ZIKV infiltration into the CNS. The outcome of ZIKV may include both a precocious differentiation of RG to astrocytes coupled with a loss of NPCs including RG due to apoptosis or autophagy has been described for neuronal stem cells in response to ZIKV [67]. In the control cortex and that of ZIKV Fetus 1 and 3, NeuN neurons were organized in long organized tracks of migration toward the CP, while in the ZIKV infected cortex, the pattern of NeuN staining was largely unorganized with a trend towards decline in NeuN positive cells in the cortical plate of the frontal cortex. This data suggests that ZIKV infection in the baboon fetal cortex at mid-gestation doesn’t affect neuronal population that migrated to form cortical layers prior to the point of infection but could still affect migration of neurons to form the final cortical layers and therefore, cortical volume at term since the SVZ in humans become the principal source of cortical neurons from 25 to 27 weeks of gestation. It should also be emphasized that the RG fibers provide an additional function in the formation of gyri and sulci [68]. Loss of RG fibers in the ZIKV brain would seemingly predict a less folded brain as gestation progresses. Loss of gyri/sulci is a hallmark in ZIKV infected cases of human microcephaly [6, 69].

**Fig 13:**
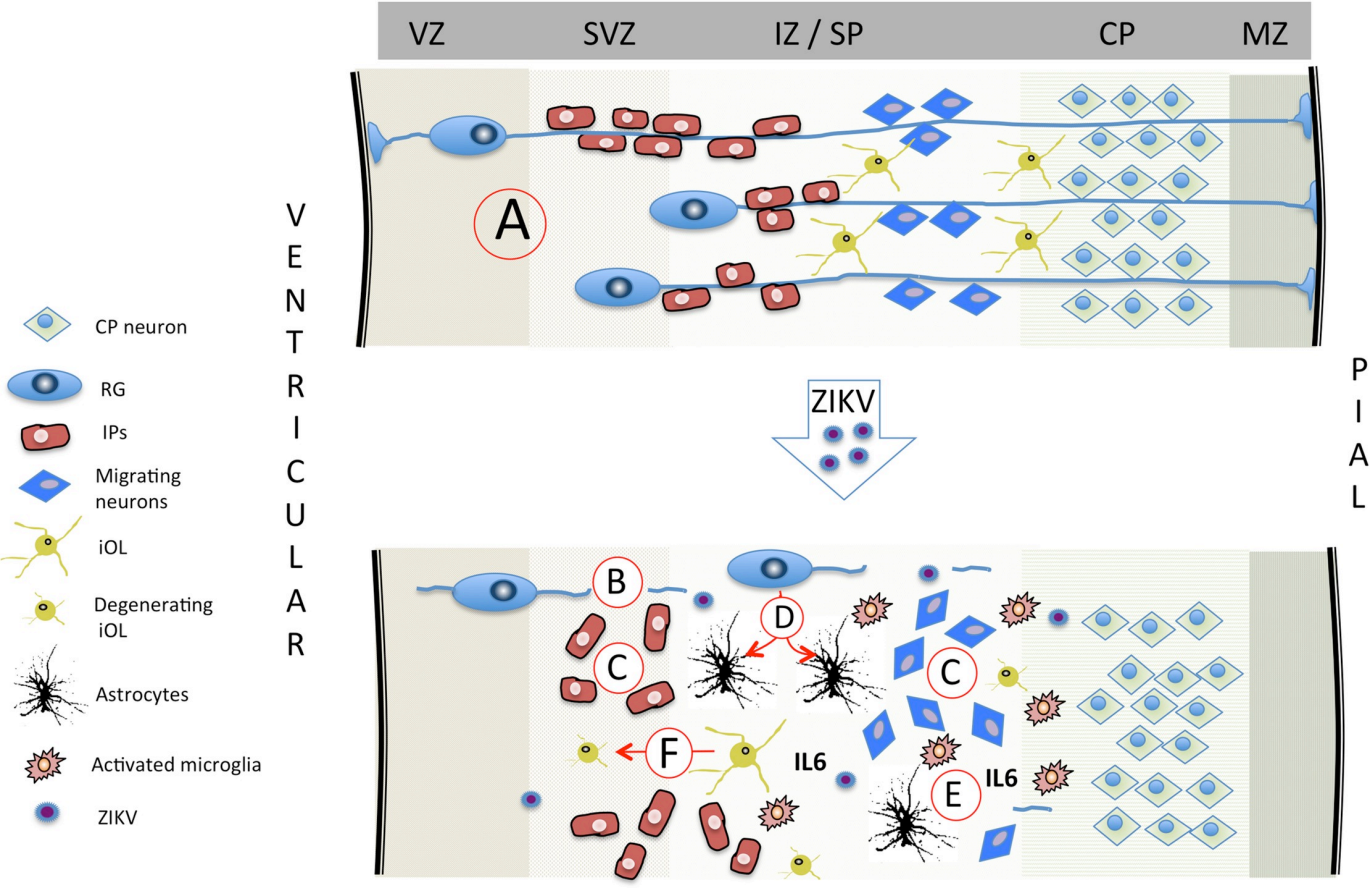
Summary diagram depicting potential mechanisms via which ZIKV may lead to fetal brain pathology in primates. (A) In the normally developing primate cortex, radial glial (RG) fibers serve as scaffolding for migrating neurons and intermediate precursors (IP) to the CP [55]. During the second half of gestation, RG end-feet detach from the ventricular and pial surfaces removing the RG fiber scaffolding after the cortex has formed its layers [56–59]. During the second half of gestation, RG also gradually differentiate to astrocytes and pre-oligodendrocytes [57–61] with normal transformation to astrocytes in the fetal frontal cortex. Based on the present study and studies in macaques (33,34,37,38), ZIKV infection of the fetal cortex results in a loss in RG fibers (B) resulting in disorganized neuroprogenitor cells (C; intermediate precursors (IP) and migrating neurons). (D) The widely reported astrogliosis may be the result of premature differentiation of RG to astrocytes, and/or as part of the noted neuroinflammatory response to ZIKV that includes increased microglia and IL-6 (E). (F) ZIKV infection impacts developing oligodendrocytes leading to reduced myelination as observed in the pigtail macaque and as reported in human fetuses [37, 38]. (Abbreviations: VZ: ventricular zone; SVZ: subventricular zone; IZ: intermediate zone; SP: subplate; CP: cortical plate; MZ: marginal zone).

In addition to increased astrocytes, the 21 day ZIKV infected cortex (Fetus 4) exhibited other indices of neuroinflammation with increased Iba1 (microglia) and IL-6 (proinflammatory cytokine) immunostaining. While astrogliosis was described in the fetal CNS of pigtail macaque fetuses when analyzed at near term gestation, there are no other reports of active neuroinflammation in studies of rhesus macaques in response to ZIKV infection. However, brain lesions have been described consisting of necrosis and gliosis in infant rhesus macaques whose mothers were infected during early gestation [34]. This study also observed vascular compromise leading to localized vascular insufficiency hemorrhage and vasculitis that via localized hypoxia/ischemia, may have played a primary role in fetal CNS pathology of these infants. Neuroinflammation in these infants could have been instrumental in synergizing with ZIKV in generating the CNS pathological outcome of these infants. Of interest was the noted increase in both Iba1 and IL-6 in the frontal cortex of the day 14 post-infection fetus (compared to the control fetus and Fetus 1) despite not detecting ZIKV in the cortex of this fetus. This implicates that the increased neuroinflammation may be in response to maternal or placental inflammation, or that ZIKV had transferred to the fetus in a tissue not sampled or in an adjacent region of the fetal brain not sampled. Increased neuroinflammation may also be a hallmark of impending ZIKV infiltration into the fetal CNS. The potential implications of increased fetal neuroinflammation in the absence of vertical transfer has implications for human infants from ZIKV infected mothers without notable gross CNS pathologies that may lead to subtle neurobehavioral or cognitive deficits post-birth. Indeed, delivery of the viral mimetic, poly (I:C) to pregnant macaques during the first or second trimesters results in offspring with notable behavioral changes reflecting autism [70]. Recently, a new study by CDC reported that 1 out of every 7 babies born from zika infected mothers in the US had birth defects or neurodevelopmental problems such as seizures and developmental delays (DOI: http://dx.doi.org/10.15585/mmwr.mm6731e1). Although it is not clear how many vertical transfers occurred in all these pregnancies, ZIKV associated neurodevelopmental abnormalities were identified in babies both positive and negative for ZIKV RNA and IgM highlighting the importance of understanding role of neuroinflammation in fetal brain in the absence of vertical transfer. Therefore, children born from mothers infected during pregnancy could suffer from long term neurodevelopmental defects despite being born without obvious brain pathology such as microcephaly. The summary of potential mechanism via which ZIKV may alter development of the primate cortex is diagrammed in Fig 13.

In conclusion, the pregnant baboon offers an additional non-human primate model for ZIKV infection and adverse pregnancy outcome to compare and contrast with macaque species. Using a moderate dose of a relevant strain of ZIKV we recapitulated both clinical signs of human ZIKV infection as well as vertical transfer and a noted cortical pathology that may provide insight into the mechanisms via which ZIKV can induce fetal CNS damage in human pregnancy. Future studies can focus on long term neurodevelopmental sequelae resulting from ZIKV infection, in particular since we observed neuroinflammation of the fetal CNS in the apparent absence of vertical transfer of the virus. This may have major ramifications for the vast majority of cases in human pregnancies infected with ZIKV without CZS yet may have significant long term cognitive and behavioral deficits in the offspring.

## Materials and methods

### Ethical statement

**Fig 14:**
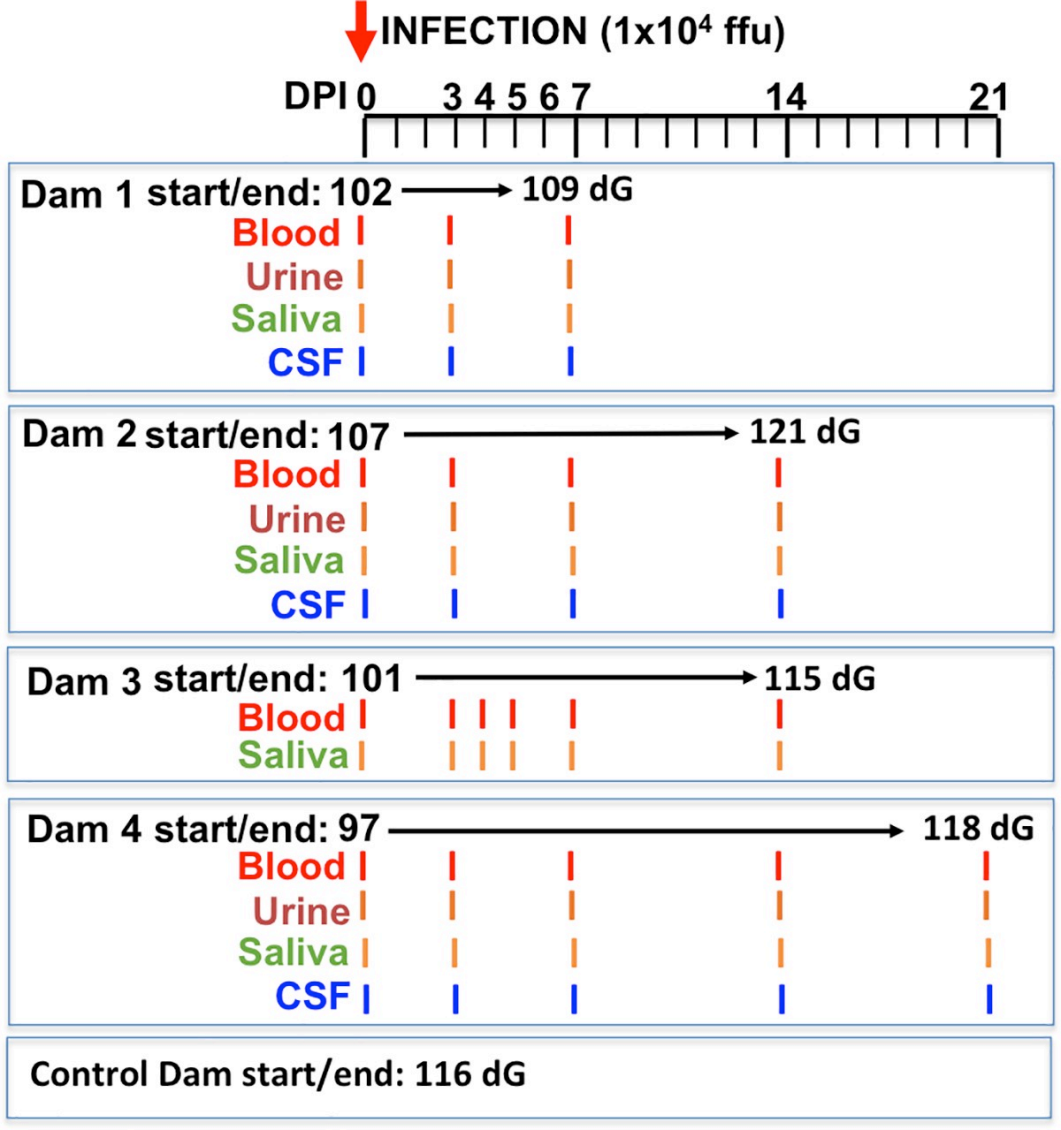
Schematic representation of the experimental design. Multiparous timed pregnant olive baboons (n = 4) were infected subcutaneously (1x10^4^ ffu, 1 ml volume, strain H/FP/2103) on Day 0. Maternal blood, urine, CSF and saliva were obtained on the indicated days for each animal. The gestational age at the time of infection ranged from 97–107 days gestation (dG). One dam was euthanized at day 7 post infection (Dam 1), two dams on day 14 post infection (Dams 2,3) and on at 21 days post infection for collection of maternal and fetal tissues. The gestational age range at necropsy was 109–121 dG; a timed pregnant control dam was euthanized at 116 dG.

All experiments utilizing baboons were performed in compliance with guidelines established by the Animal Welfare Act for housing and care of laboratory animals and conducted in accordance with and approval from the University of Oklahoma Health Sciences Center Institutional Animal Care and Use Committee (IACUC; protocol no. 101523-16-039-I). All studies with ZIKV infection were performed in Assessment and Accreditation of Laboratory Animal Care (AAALAC) International accredited ABSL2 containment facilities at the OUHSC. Baboons were fed standard monkey chow twice daily as well as receiving daily food supplements (fruits). Appropriate measures were utilized to reduce potential distress, pain and discomfort, including post-CSF collection analgesia. All animals received environmental enrichment. ZIKV infected animals were caged separately but within visual and auditory contact of other baboons to promote social behavior and alleviate stress. At the designated times post inoculation (Fig 14), the animals were euthanized according to the recommendations of the American Veterinary Medical Association (2013 panel on Euthanasia).

### Animals

Adult timed pregnant female olive baboons (n = 5, 6–15 years of age) were utilized for this study. All females were multiparous with history of successful prior pregnancies. All dams used in this study were determined to be seronegative for West Nile Virus [25].

#### Virus stocks, infection and sample collection

Animals were anaesthetized with an intramuscular dose of Ketamine (10 mg/kg) before all procedures (viral inoculation, blood, salivary and vaginal swabs and urine collection). Timed pregnant female baboons were infected subcutaneously at the mid-scapular area with a single clinically relevant dose of 10^4^ focus forming units (ffu; 1 ml volume per dose) of the French Polynesian ZIKV isolate (H/PF/2013). The dosage used to infect the animals in our study is based on the previous works done in mosquitoes carrying WNV and DENV, where it was estimated that mosquitoes carry 1x10^4^ to 1x10^6^ plaque forming units (PFU) of the virus [71], from a study evaluating Brazilian ZIKV in a bite from *Aedes aegypti* mosquito[72] and from a study of mosquito transmission of ZIKV in rhesus monkeys [73]. The pregnant females were infected near mid-gestation (between 97 and 107 days of gestation [dG]; term is approx. 181 dG; the overall approach is detailed in Fig 14). Maternal blood samples, vaginal and salivary swabs and urine was obtained on the day of inoculation (day 0) as well as ultrasound evaluation of fetal viability. Whole blood was collected into EDTA tubes. Urine was collected by direct bladder cystocentesis. Saliva and vaginal samples were collected by cotton roll salivette. The sampling procedure for each dam is detailed in Fig 1. For Dam 1, blood, urine, vaginal, salivary and CSF samples were obtained at 3 dpi and at 7 dpi, immediately prior to euthanasia at 7 dpi and maternal-fetal tissue collection. For Dam 2, blood, urine, vaginal, salivary and CSF samples were collected at 3 dpi and 7 dpi and at 14 dpi, immediately prior to euthanasia (14 dpi) and maternal-fetal tissue collection. For Dam 3 blood, vaginal, salivary samples were collected on days 3,4,5 and 7 dpi and at 14 dpi, immediately prior to euthanasia (14 dpi) and maternal-fetal tissue collection (no urine or CSF collection for this Dam). For Dam 4, blood, urine, vaginal, salivary and CSF samples were collected at 3, 7and 14 dpi, and at 21 dpi, immediately prior to euthanasia (21 dpi) and maternal-fetal tissue collection. A control timed pregnant dam was euthanized on 116 dG.

At the end of the study for each animal, dams were sedated with ketamine, all maternal samples obtained as well as ultrasound measurements, then the animal rapidly euthanized with euthasol. A C-section was quickly performed, cord blood obtained and the fetus euthanized with euthasol. Maternal and fetal tissues were rapidly collected and samples were both fixed with 4% paraformaldehyde and frozen on dry ice (stored at—80°C) for each tissue.

Complete blood counts (CBCs): CBCs were evaluated for all females on EDTA-anticoagulated whole blood samples collected on day 0 and subsequent days-post infection as shown in the experimental timeline (Idexx ProCyte DX hematology analyzer; Idexx laboratories, ME). CBC’s included analysis for red blood cells (RBCs), hemoglobin, hematocrit and platelet count. RBC, hemoglobin and hematocrit numbers did not show any differences pre-and post ZIKV infection for any of the infected females. Platelet counts did not change in response to ZIKV infection in any dam.

#### One-Step quantitative reverse transcription PCR

Primers and probes used for qRT-PCR were designed by Lanciotti et al [74] (**Table 2**). RNA was isolated from maternal and fetal tissues (**Table 1**) using QIAamp cador pathogen mini kit (Qiagen, Valencia, CA). ZIKV RNA was quantitated by one-step quantitative real time reverse transcription PCR using QuantiTect probe RT-PCR kit (Qiagen) on an iCycler instrument (BioRad). Primers and probes were used at a concentration of 0.4 μM and 0.2 μM respectively and cycling conditions used were 50°C for 30 min, 95°C for 15 min followed by 40 cycles of 94°C for 15 s and 60°C for 1 min. Concentration of the viral RNA (copies/milliliter) was determined by interpolation onto a standard curve of six 10-fold serial dilutions (10^6^ to 10^1^ copies/ml)) of a synthetic ZIKV RNA fragment available commercially from ATCC (ATCC VR-3252SD). The cutoff for limit of detection of ZIKV RNA was 1x10^2^.

**Table 2:** Primer/Probe sets for the detection of ZIKV by one step qRT-PCR.

	Primers	Genome Position	Sequence (5`-3`)
1	ZIKV 835 Forward	835–857	TTGGTCATGATACTGCTGATTGC
	ZIKV 911 Reverse	890–911	CCTTCCACAAAGTCCCTATTGC
	ZIKV 860-FAM Probe	860–886	CGGCATACAGCATCAGGTGCATAGGAG
2	ZIKV 1086 Forward	1086–1102	CCGCTGCCCAACACAAG
	ZIKV 1162 Reverse	1162–1139	CCACTAACGTTCTTTTGCAGACAT
	ZIKV 1107-FAM Probe	1107–1137	AGCCTACCTTGACAAGCAGTCAGACACTCAA

#### ZIKV ELISA

ZIKA specific IgM and IgG antibody responses were assessed in the serum samples using the commercially available anti-ZIKV IgM (#ab213327, Abcam, Cambridge, MA) and IgG (#Sp856C, XpressBio, Fredrick, MD) ELISA kits. Briefly, a 1:100 for IgM and 1:50 for IgG serum dilution was performed in duplicate and added to the pre-coated plates available in the kits. The assays were performed using the manufacturer’s instructions and the assay was read at 450 nm for IgM and 405 nm for IgG antibodies in the serum.

#### Plaque Reduction Neutralization Test (PRNT)

A plaque reduction neutralization test (PRNT) was used to assess serum samples for ZIKV neutralizing antibodies. Vero cells (ATCC #CCL-81) were maintained in DMEM (supplemented with 10% heat-inactivated FBS, 1x antibiotic/antimycotic), seeded in 12-well plates (2 x 10^5^ cells/well, 37^o^ C, 5% CO_2_) and incubated at for approximately 24 hours until 80–100% confluent. Baboon sera were heat inactivated (56^o^ C, 30 min) then serially diluted 2-fold in media, followed by addition of 200 plaque forming units (pfu) of French Polynesian ZIKV isolate (H/PF/2013). Samples were vortexed and incubated in a 37^o^ C water bath for one hour. Media was then removed from each well and replaced with the virus/serum mixture followed by incubation at 37^o^ C for one hour with intermittent rocking of the plates every 20 minutes. Control wells included inoculation with 200 pfu of ZIKV with no serum added to determine total plaques, as well as control wells without virus. A 1% carboxymethyl cellulose overlay was then added without removing the inoculum and the plates were incubated at 37^o^ C for approximately 72 hours. The cells were then fixed with 1% paraformaldehyde for one hour, after which, the overlay was removed and the cells were stained with 1% crystal violet. The PRNT50, or the concentration of serum required to neutralize 50% of the plaque count of a known amount of serum-free ZIKV, was calculated by visually counting plaques.

#### Serum cytokine analysis

Non-human primate cytokine/chemokine/inflammatory panel V-PLEX Multi-Spot assay system (Meso Scale Discovery, Rockville, MD) was used to quantify 24 cytokines and chemokines from plasma obtained from dams and cord blood. The assays were performed according to the manufacturer’s instructions. Briefly, plasma samples were diluted 2-fold (inflammatory, cytokine panel) and 4-fold (chemokine panel) in respective diluents and added in duplicates to plates for each panel and incubated overnight on a shaker at 4°C. Plates were washed 3 times and detection antibody cocktail specific for each panel was added to the respective plates and incubated for 2 hours at room temperature on a shaker. Plates were washed and read using an MSD instrument and the final data was obtained using the MSD discovery workbench software.

#### Fetal CNS and placental immunohistochemistry/immunofluorescence

**Fig 15:**
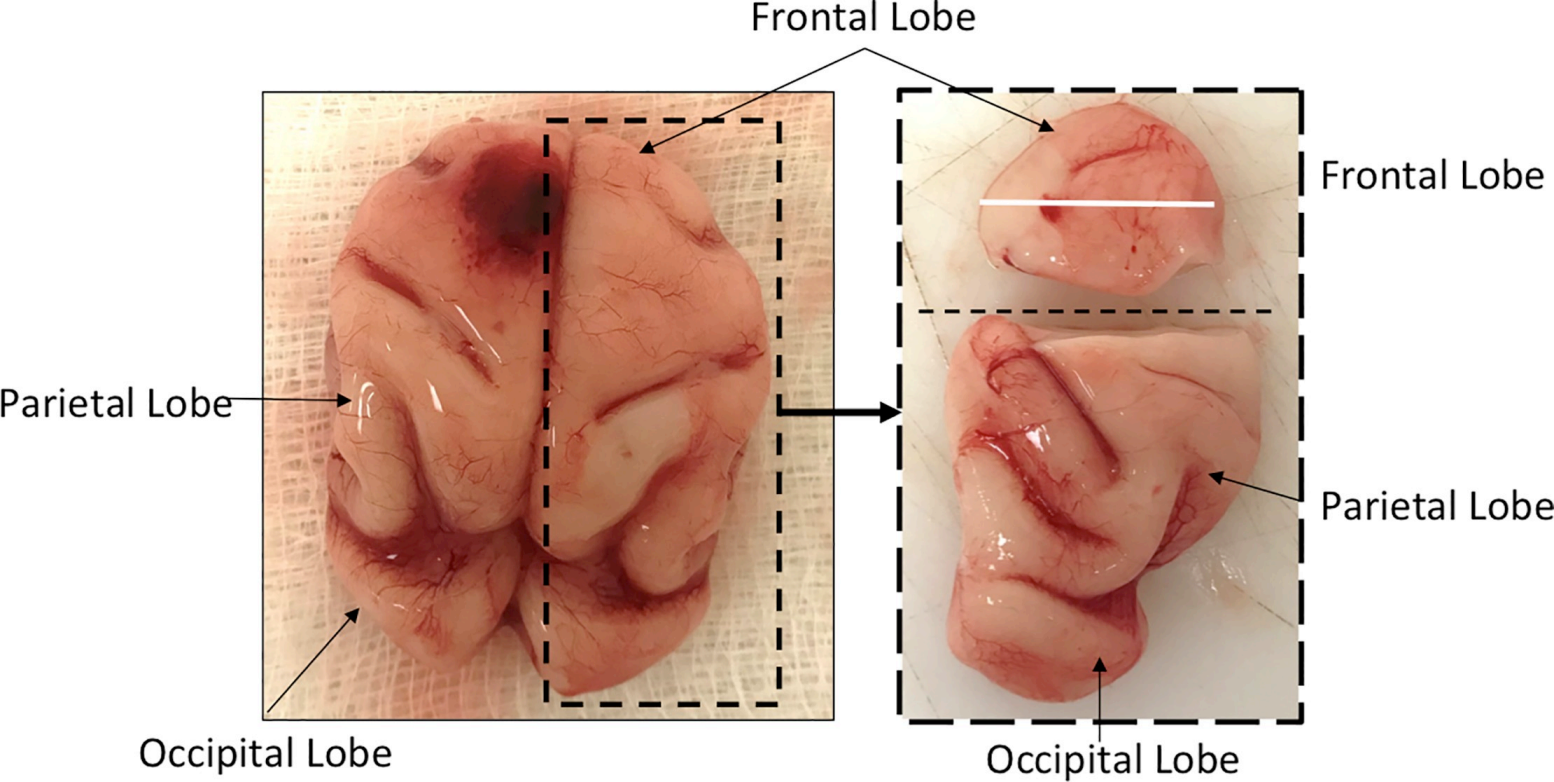
120 day gestation fetal baboon brain depicting the lobes and location of the sections used for immunohistochemical analysis (white line in frontal cortex).

Following removal, fetal brains were divided mid-sagittal with one half rapidly frozen on dry ice and stored at -80°C and the other half fixed in 4% paraformaldehyde for 24 hours, cryoprotected in 30% sucrose until sunk and frozen in embedding molds in optimal cutting temperature (OCT, Sakura Finetek) on dry ice and stored at -80°C. The identical region of the frontal cortex (Fig 15) were selected from each fetus and subjected to serial coronal sectioning (30μm) on a freezing microtome and collected in 24 well plates in a cryoprotectant solution at -20°C. Routine Nissl staining was performed initially to ascertain that each frontal cortex was being sectioned in the same plane and region for analysis. For immunocytochemical and immunofluorescent labelling, every fifth free-floating section (every 150 microns) were selected for immunolabeling for a total of 3 sections per fetus.

For Iba-1 and IL-6 immunohistochemistry, sections were treated with 2% H_2_O_2_ solution to inactivate endogenous peroxidase activity followed by blocking and primary antibody incubation overnight at room temperature with rocking (Iba-1; Rabbit polyclonal 1:100, NBP2-16901; IL-6; Rabbit polyclonal 1:200, NB600-1131; NovusBio, Littleton, CO). Sections were washed and incubated with goat anti-rabbit HRP conjugated secondary antibody for 1 h at RT. Immunolabeling was visualized using DAB substrate and sections were washed and dried overnight before a cover slip was added with Permount (FisherScientific, Fairlawn, NJ). Control reactions were performed where the primary antibody was omitted from the procedure. Sections were visualized using a brightfield microscope (Olympus B40x) equipped with a SPOT 5 MP digital camera with SPOT 5.3 imaging software (Sterling Heights, MI).

For single and dual Immunofluorescence labeling (IF), sections were treated with 1% NaBH4 solution to reduce auto-fluorescence then blocked for an hour in blocking solution followed by overnight incubation in single or double primary antibodies (GFAP; mouse monoclonal 1:1000, NBP1-05197; Nestin; chicken polyclonal 1:1000, NB100-1604, Novus Bio, Littleton, CO; NeuN, mouse monoclonal 1:100, MAB377, Millipore, Temecula, CA; O1, mouse monoclonal 1:20, MAB1327, Bio-Techne, Minneapolis, MN, Pan anti-flavivirus; mouse monoclonal 1:500, MAB10216, Millipore, CA). Sections were incubated for an hour the next day at room temperature in goat anti-mouse Alexa 568, Alexa 598 and goat anti-chicken Alexa 488 1:200 (Life Technologies, Carlsbad, CA). Sections were washed in PBS and transferred to slides, incubated for 30 min in dark at room temperature in Prolong Gold with DAPI (Life technologies, Carlsbad,CA), cover slipped and cured for 24 h before visualizing using fluorescent microscope (Zeiss Axiovert 200 inverted fluorescent microscope). Controls again were exclusion of the primary antibodies. Images were captured using AxioVision imaging software (Zeiss, Germany).

#### Image analysis

Image analysis was performed using NIH ImageJ (Wayne Rasband, NIH) based in part on our published methods for quantification of neuronal numbers and structural volume in the CNS ([75, 76]). Original ICC or IF images were deconvoluted using ImageJ to separate individual colors (Red, green, blue) using identical parameters for each section (minimum, maximum, brightness), then for each color, converted to 16 bit greyscale images. Images were then adjusted by thresholding using identical parameters (range of pixel intensities for white and black) for each image, background was subtracted using the rolling ball method then processed using Binary/Watershed to separate objects (e.g. labelled cells touching) then counted using analyze particles (cell counts). This method was found by a blinded observer to produce the same cell counts per area as manually counting labelled cells/neurons. For RG fibers, astroctyes/RG bodies were identified by thresholding and deleting from the images and the remaining fibers digitally counted (pixels at 0/black). Cortical volume analysis was performed as previously described in our laboratory.

#### TUNEL assay (apoptosis)

Terminal deoxynucleotidyl transferase dUTP nick end labeling (TUNEL) assay was performed using a commercially available kit from Trevigen (Gaithersburg, MD). Briefly, fixed frozen fetal cortical sections were washed with PBS and incubated with Cytonin for 30 min, washed, and quenched before labeling with biotin-labeled dUTP. The labeling reaction was stopped by adding stop buffer. The tissue sections were then incubated with HRP-conjugated streptavidin for 10 min, washed, and immersed in DAB solution for color development. Sections were counterstained with methyl green before mounting. A positive and a negative control sections were included in the protocol. Apoptotic cells are stained with dark brown color.
